# Supplementary material for: Interpretable deep cross networks unveiled common signatures of dysregulated epitranscriptomes across 12 cancer types
Source: Mol Ther Nucleic Acids. 2024 Oct 29;35(4):102376. doi: 10.1016/j.omtn.2024.102376 (PMC11605186; doi:10.1016/j.omtn.2024.102376)
Supplement: Document S1. Figures S1–S3 and Tables S1–S3, S5, S6, and S13 [file mmc1.pdf]

OMTN, Volume *35*

Supplemental information

Interpretable deep cross networks unveiled common signatures of dysregulated epitranscriptomes across 12 cancer types

Rong Xia, Xiangyu Yin, Jiaming Huang, Kunqi Chen, Jiongming Ma, Zhen Wei, Jionglong Su, Neil Blake, Daniel J. Rigden, Jia Meng, and Bowen Song

Table S1. Performance evaluation using different sequence-based feature extraction approaches (AUROC)

	One-hot	K-mer counting	PSSMs
Lung cancer	0.56	0.50	0.52
Bladder cancer	0.61	0.60	0.59
Colon cancer	0.55	0.57	0.56
Brain cancer	0.52	0.50	0.52
Kidney cancer	0.51	0.50	0.52
Prostate cancer	0.59	0.58	0.50
Bone marrow cancer	0.73	0.50	0.60
Liver cancer	0.58	0.59	0.50
Ovary cancer	0.55	0.49	0.50
Lymph nodes cancer	0.78	0.64	0.59
Skin cancer	0.55	0.51	0.50
Stomach cancer	0.64	0.60	0.59

Table S2. Performance evaluation of sequence-only, genomic-only and integration models

	Sequence only model	Genomic only model	Integration model
Liver cancer	0.58	0.73	0.76
Ovary cancer	0.55	0.70	0.72
Bone marrow cancer	0.73	0.84	0.86
Bladder cancer	0.61	0.73	0.74
Colon cancer	0.55	0.83	0.84
Brain cancer	0.52	0.87	0.87
Kidney cancer	0.51	0.61	0.62
Prostate cancer	0.59	0.75	0.76
Lung cancer	0.56	0.82	0.81
Lymph nodes cancer	0.78	0.86	0.86
Skin cancer	0.55	0.78	0.78
Stomach cancer	0.64	0.83	0.81

Table S3. Performance evaluation of 12 cancer type-specific model

	Independent testing					
	Sn (%)	Sp (%)	ACC (%)	MCC	F1	AUROC
Lung cancer	0.77	0.68	0.73	0.74	0.46	0.81
Bladder cancer	0.75	0.59	0.67	0.70	0.34	0.74
Colon cancer	0.81	0.68	0.74	0.76	0.49	0.84
Brain cancer	0.80	0.75	0.78	0.78	0.55	0.87
Kidney cancer	0.51	0.61	0.56	0.54	0.12	0.62
Prostate cancer	0.62	0.68	0.65	0.64	0.29	0.76
Bone marrow cancer	0.77	0.76	0.76	0.76	0.53	0.86
Liver cancer	0.77	0.65	0.70	0.72	0.40	0.76
Ovary cancer	0.63	0.65	0.64	0.64	0.28	0.72
Lymph nodes cancer	0.75	0.77	0.76	0.76	0.53	0.86
Skin cancer	0.71	0.70	0.71	0.71	0.41	0.78
Stomach cancer	0.76	0.78	0.77	0.77	0.54	0.86

Table S4. Performance of cancer type-cross testing (AUROC)

Table S5. Performance evaluation of sequence-only input using different model settings (AUROC)

Activation Function	Two layers			Three layers			Five layers			Seven layers			10 layers		
	10 epoch	20 epoch	50 epoch	10 epoch	20 epoch	50 epoch	10 epoch	20 epoch	50 epoch	10 epoch	20 epoch	50 epoch	10 epoch	20 epoch	50 epoch
Sigmoid	0.54	0.52	0.51	0.54	0.52	0.50	0.54	0.52	0.50	0.53	0.52	0.52	0.52	0.54	0.51
ReLU	0.53	0.55	0.54	0.55	0.51	0.52	0.55	0.53	0.53	0.55	0.52	0.52	0.54	0.53	0.50
Tanh	0.54	0.52	0.52	0.54	0.50	0.51	0.52	0.52	0.50	0.54	0.52	0.50	0.54	0.50	0.52
LeakyReLU	0.50	0.51	0.53	0.53	0.50	0.52	0.54	0.52	0.52	0.52	0.51	0.52	0.55	0.52	0.53

Table S6. Performance evaluation using different algorithms

	DCN	Simple CNN	Logistic Regression	Random Forest	Gradient Boosting
Lung cancer	0.81	0.78	0.74	0.83	0.79
Bladder cancer	0.74	0.70	0.69	0.75	0.73
Colon cancer	0.84	0.78	0.76	0.82	0.79
Brain cancer	0.87	0.80	0.78	0.83	0.81
Kidney cancer	0.62	0.56	0.52	0.74	0.67
Prostate cancer	0.76	0.69	0.64	0.76	0.69
Bone marrow cancer	0.86	0.79	0.75	0.85	0.79
Liver cancer	0.76	0.70	0.63	0.83	0.68
Ovary cancer	0.72	0.67	0.61	0.74	0.69
Lymph nodes cancer	0.86	0.81	0.79	0.81	0.80
Skin cancer	0.78	0.71	0.66	0.79	0.74
Stomach cancer	0.86	0.61	0.78	0.83	0.81
Average	0.80	0.72	0.70	0.80	0.75

Table S7. Gene Ontology (GO) enrichment analysis of the most cancers-associated m6A sites involved in pan-cancer landscape

Table S8. Kyoto Encyclopedia of Genes and Genomes (KEGG) analysis of the most cancers-associated m6A sites involved in pan-cancer landscape

Table S9. m6A-MeRIP-seq samples

Table S10. Cancer type-specific dataset

Table S11. Cancers-associated m6A sites used in developing pan-cancer model

Table S12. Normal tissues m6A sites used in developing pan-cancer model

Table S13. Genomic knowledges considered in the study

<table>
<tr><td>ID</td><td>Name</td><td>Description</td><td>Note</td></tr>
<tr><td>1</td><td>UTR5</td><td>5' UTR</td><td rowspan="13">Dummy variables indicating whether the site is overlapped to the topological region on the major RNA transcript.</td></tr>
<tr><td>2</td><td>UTR3</td><td>3' UTR</td></tr>
<tr><td>3</td><td>cds</td><td>Coding sequence</td></tr>
<tr><td>4</td><td>Stop_codons</td><td>stop codons flanked by 100bp</td></tr>
<tr><td>5</td><td>Start_codons</td><td>start codons flanked by 100bp</td></tr>
<tr><td>6</td><td>TSS</td><td>downstream 100bp of TSS</td></tr>
<tr><td>7</td><td>TSS_A</td><td>downstream 100bp of TSS on A</td></tr>
<tr><td>8</td><td>exon_stop</td><td>exons containing stop codons</td></tr>
<tr><td>9</td><td>alternative_exon</td><td>alternative exons</td></tr>
<tr><td>10</td><td>constitutive_exon</td><td>constitutive exons</td></tr>
<tr><td>11</td><td>internal_exon</td><td>Internal exons</td></tr>
<tr><td>12</td><td>long_exon</td><td>long exons (exon length >= 400bp)</td></tr>
<tr><td>13</td><td>last_exon</td><td>5’ last_exon</td></tr>
<tr><td>14</td><td>length_UTR3</td><td>3'UTR length</td><td rowspan="6">The region length in bp</td></tr>
<tr><td>15</td><td>length_UTR5</td><td>5'UTR length</td></tr>
<tr><td>16</td><td>length_cds</td><td>coding sequence length</td></tr>
<tr><td>17</td><td>length_tx_full</td><td>full transcript length</td></tr>
<tr><td>18</td><td>length_gene_full</td><td>full gene length</td></tr>
<tr><td>19</td><td>length_gene_ex</td><td>length of all exons of the gene</td></tr>
<tr><td>20</td><td>clust_f1000</td><td>count of neighboring input site at 1001 bp</td><td rowspan="6">Clustering information</td></tr>
<tr><td>21</td><td>clust_f100</td><td>count of neighboring input site at 101 bp</td></tr>
<tr><td>22</td><td>clust_A_f1000</td><td>count of neighboring A within in 2001 nt window</td></tr>
<tr><td>23</td><td>clust_A_f100</td><td>count of neighboring A within 201 nt window</td></tr>
<tr><td>24</td><td>dist_nearest_p2000</td><td>distance to the closest neighboring input site at 2001 bp</td></tr>
<tr><td>25</td><td>dist_nearest_p200</td><td>distance to the closest neighboring input site at 201 bp</td></tr>
<tr><td>26</td><td>PC_1bp</td><td>phastCons scores of the nucleotide</td><td rowspan="4">Scores related to evolutionary conservation</td></tr>
<tr><td>27</td><td>PC_101bp</td><td>average phastCons scores within the flanking 101 bp</td></tr>
<tr><td>28</td><td>FC_1bp</td><td>fitCons scores of the nucleotide</td></tr>
<tr><td>29</td><td>FC_101bp</td><td>average fitCons scores within the flanking 101 bp region</td></tr>
<tr><td>30</td><td>struct_hybridize</td><td>predicted RNA hybridized region</td><td rowspan="2">RNA secondary structures</td></tr>
<tr><td>31</td><td>struct_loop</td><td>predicted RNA loop region</td></tr>
<tr><td>32</td><td>sncRNA</td><td>sncRNA</td><td rowspan="5">Attributes of the genes or transcripts</td></tr>
<tr><td>33</td><td>lncRNA</td><td>lncRNA</td></tr>
<tr><td>34</td><td>HK_genes</td><td>housekeeping genes</td></tr>
<tr><td>35</td><td>miR_targeted_genes</td><td>miRNA targeted genes</td></tr>
<tr><td>36</td><td>HNRNPC_eCLIP</td><td>eCLIP data of HNRNPC RNA binding sites</td></tr>
</table>

37	TargetScan	predicted miRNA targeted sites by TargetScan	
38	Verified_miRtargets	miRNA targeted sites verified by experiment	
39	METTL3_TREW	overlapped with binding regions of METTL3	
40	METTL14_TREW	overlapped with binding regions of METTL14	
41	WTAP_TREW	overlapped with binding regions of WTAP	
42	METTL16_CLIP	overlapped with binding regions of METTL16	
43	ALKBH5_PARCLIP	overlapped with binding regions of ALKBH5	
44	FTO_CLIP	overlapped with binding regions of FTO	
45	isoform_num	number of isoforms	Genomic properties
46	exon_num	number of exons	
47	GC_cont_genes	GC composition of genes	
48	GC_cont_101bp_abs	GC composition of 101 bp	
49	pos_UTR5	relative position on 5'UTR	Relative position on the region
50	pos_UTR3	relative position on 3'UTR	
51	pos_cds	relative position on coding sequence	
52	pos_exons	relative position on exon	

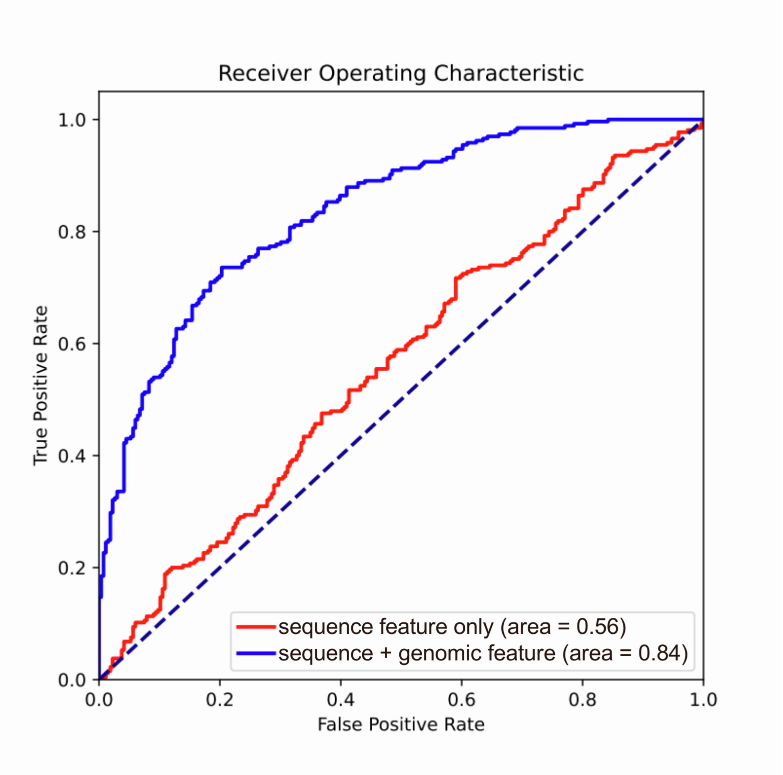

Figure S1. Performance evaluation of m^{6}A pan-cancer model (AUROC).

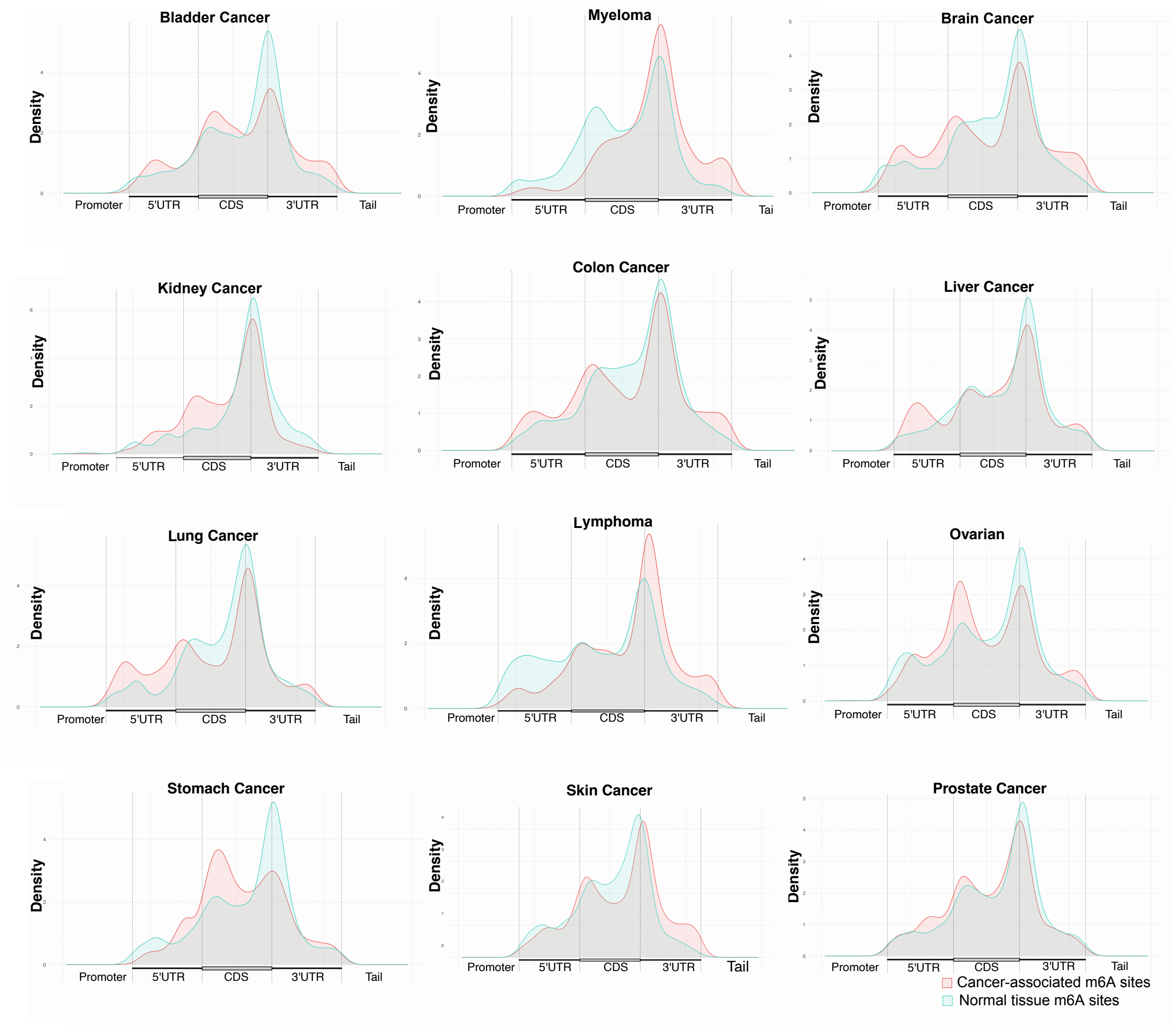

Figure S2. Distribution of 12 types of cancer type-specific m^{6}A modification sites on mRNA.

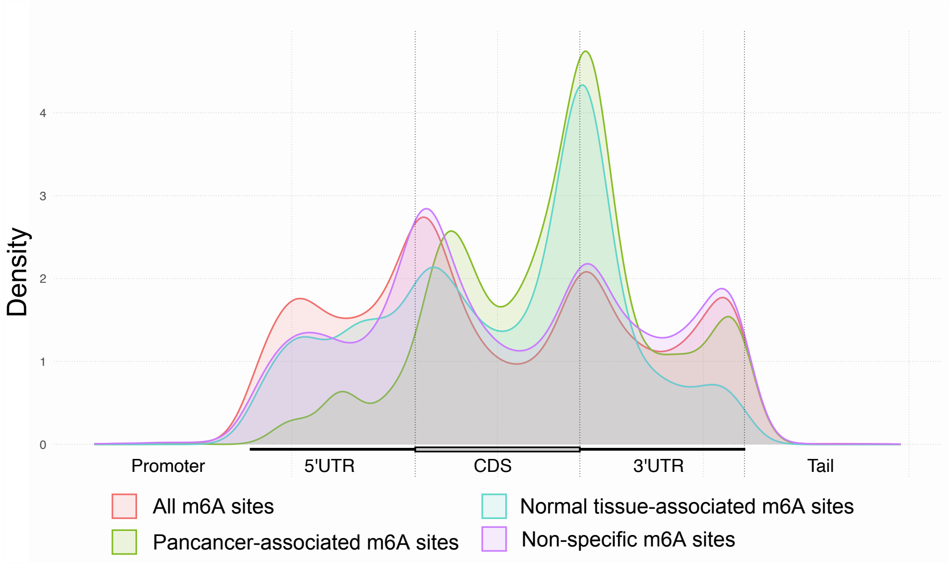

Figure S3. Distribution of pancancer-associated, normal tissue-associated, non-specific, and all m^{6}A methylation sites on mRNA.
