# Supplementary material for: Interpretable deep cross networks unveiled common signatures of dysregulated epitranscriptomes across 12 cancer types
Source: Mol Ther Nucleic Acids. 2024 Oct 29;35(4):102376. doi: 10.1016/j.omtn.2024.102376 (PMC11605186; doi:10.1016/j.omtn.2024.102376)
Supplement: Document S2. Article plus supplemental information [file mmc9.pdf]

Molecular Therapy
Nucleic Acids
Original Article

Interpretable deep cross networks unveiled common signatures of dysregulated epitranscriptomes across 12 cancer types

Rong Xia,[1,3,4,8] Xiangyu Yin,[3,7,8] Jiaming Huang,[3] Kunqi Chen,[5] Jiongming Ma,[3] Zhen Wei,[3,6] Jionglong Su,[4] Neil Blake,[7] Daniel J. Rigden,[7] Jia Meng,[2,3,7] and Bowen Song[1]

[1]Department of Public Health, School of Medicine, Nanjing University of Chinese Medicine, Nanjing 210023, China; [2]Institute of Biomedical Research, Regulatory Mechanism and Targeted Therapy for Liver Cancer Shiyan Key Laboratory, Hubei Provincial Clinical Research Center for Precise Diagnosis and Treatment of Liver Cancer, Taihe Hospital, Hubei University of Medicine, Shiyan, Hubei 442000, China; [3]Department of Biological Sciences, School of Science, Suzhou Key Laboratory of Cancer Biology and Chronic Disease, Xi'an Jiaotong-Liverpool University, Suzhou 215123, China; [4]School of AI and Advanced Computing, XJTLU Entrepreneur College (Taicang), Xi'an Jiaotong-Liverpool University, Suzhou, Jiangsu 215123, China; [5]Key Laboratory of Ministry of Education for Gastrointestinal Cancer, School of Basic Medical Sciences, Fujian Medical University, Fuzhou 350004, China; [6]Institute of Infection, Veterinary & Ecological Sciences, University of Liverpool, L7 8TX Liverpool, UK; [7]Institute of Systems, Molecular and Integrative Biology, University of Liverpool, L7 8TX Liverpool, UK

Cancer is a complex and multifaceted group of diseases characterized by uncontrolled cell growth that leads to the formation of malignant tumors. Recent studies suggest that N6-methyladenosine (m^{6}A) RNA methylation plays pivotal roles in cancer pathology by influencing various cellular processes. However, the degree to which these mechanisms are shared across different cancer types remains unclear. In this study, we analyze an expansive array of 167 m^{6}A epitranscriptome profiles covering 12 distinct cancer types and their originating normal tissues. We trained 12 distinct, cancer type-specific interpretable deep cross network models, which successfully distinguish between specific pairs of normal and cancer m^{6}A contexts using integrated information from both the sequences and curated genomic knowledge. Interestingly, cross-cancer type testing indicated the existence of shared genomic patterns across various cancers at the epitranscriptome level. A pan-cancer model was subsequently developed to identify these shared patterns that could not be observed in a single cancer type. Our analysis uncovered, for the first time, a common epitranscriptome signature shared across multiple cancer types, particularly associated with RNA hybridization process and aberrant splicing. This highlights the importance of a comprehensive understanding of the pan-cancer epitranscriptome and holding potential implications in the development of RNA methylation-based therapeutics for various cancers.

INTRODUCTION

Our understanding of cancer biology has been continuously broadening through systematic studies that focus on both genetic and epigenetic alterations in oncogenes, shedding light on the intricate molecular mechanisms that drive tumorigenesis.[1,2] Accumulating evidence has indicated a strong effect of RNA methylation on tumor initiation and cancer progression,[3–7] mostly for the well-studied N6-methyladenosine (m^{6}A) methylome. In human disease, m^{6}A dysregulation has been reported to play an essential role in tumor proliferation, migration, and invasion across different cancer types,[8,9] including breast cancer,[10,11] lung cancer,[12] bladder tumors,[13,14] and liver cancer.[15,16] However, the function and mechanism of m^{6}A methylation in regulating many tumor processes still remain poorly characterized, especially on the key question of whether multiple cancer types share commonalities at the epitranscriptomic layer.

Previous pan-cancer analysis has successfully identified shared post-translational modifications patterns of protein regulation, such as pan-cancer patterns of changes in protein acetylation and protein phosphorylation.[17] In addition, efforts have also been made to explore the relationship between cancer genomics and transcriptomics, which typically focus on changes in gene expression or splicing,[18] mRNA-protein correlations,[19] potential biomarker of RNA modification (RM) regulators (e.g., ALKBH5 and YTHDF1),[20,21] RM regulators and their interacting RNAs,[22] and RM-mediator genes.[23] These studies together have expanded our knowledge on cancer biology through different regulatory networks. Nevertheless, to the best of our knowledge, none of these pioneer studies have directly examined the pan-cancer epitranscriptome disturbance, while indirect study of the epitranscriptome by examining its mediator can be less reliable due to possible RM-independent functions[24,25] and complex

Received 31 March 2024; accepted 25 October 2024;
https://doi.org/10.1016/j.omtn.2024.102376.

[8]These authors contributed equally

Correspondence: Jia Meng, Department of Biological Sciences, School of Science, Suzhou Key Laboratory of Cancer Biology and Chronic Disease, Xi'an Jiaotong-Liverpool University, Suzhou 215123, China.
E-mail: jia.meng@xjtlu.edu.cn
Correspondence: Bowen Song, Department of Public Health, School of Medicine, Nanjing University of Chinese Medicine, Nanjing 210023, China.
E-mail: bowen.song@njucm.edu.cn

© 2024 The Author(s).
Published by Elsevier Inc. on behalf of The American Society of Gene and Cell Therapy.
This is an open access article under the CC BY license (http://creativecommons.org/licenses/by/4.0/).

context-specific regulations.[26] Given that DNA methylation plays a critical role in the development of DNA methylation-based cancer biomarkers,[27] it is equally important to carefully examine the genomic contexts of RNA epitranscriptome disturbance across various cancer types. This is particularly crucial considering that traditional RM-cancer analysis in general, has predominantly focused on examining the impact of hyper- or hypomethylation of RM-associated regulators.

The combination of a deep learning model and an interpretation method has been applied to address diverse biological problems[28] including phenotype prediction,[29] transcriptome signature identification,[18] cell-specific prediction of eukaryotic origins of replication sites,[30] crosstalk among different types of RMs,[31] and context-specific profiling of m^{6}A residues.[32] The combination allows for clarification of the key features driving the model decisions alongside obtaining accurate prediction results. Consequently, the detailed biological mechanisms underlying the model task can be better understood.

Here, we leveraged a deep cross networks (DCN) model with high interpretation capability to unveil shared genomic patterns of m^{6}A methylome across 12 human cancer types. The analysis is based on a large array of m^{6}A-MeRIP-seq profiling datasets derived from cancer cell lines and their corresponding tumor-originating normal tissues. We first trained 12 distinct cancer type-specific deep cross network models that successfully distinguish between a specific pair of normal and cancer m^{6}A signals. Interestingly, we found that cancer-induced m^{6}A alterations exhibit significant genomic signatures and are shared across different cancer types, suggesting that shared patterns of epitranscriptomic disturbance may exist across cancers. To this end, we then generated a harmonized pan-cancer model using data critically selected from 23 cancer cell lines, encompassing samples from 167 m^{6}A-MeRIP-seq datasets. This allowed us to explore the shared patterns that could not be identified in a single cancer type. Our pan-cancer analysis revealed that specific genomic loci, the RNA hybridization process, and aberrant splicing are features on which m^{6}A disturbance might act across a diverse range of cancer types. In addition, we performed the functional characterization of the pan-cancer-associated m^{6}A methylome.

Overall, we presented here the first pan-cancer study that details the potential regulatory mechanisms of m^{6}A methylation and their shared signatures across 12 cancer types (see Figure 1). Our findings provide new insights into cancer biology, particularly in understanding regulation through the epitranscriptome layer, with further implications for the development of RNA methylation-based diagnostic and predictive biomarkers.

RESULTS

Changes in m^{6}A methylation in cancer cannot be effectively distinguished at the sequence level

We aimed to identify the driving signatures behind the m^{6}A dynamics in cancer. Firstly, we extracted just the sequence characteristics for model development and tested their performance for each cancer type. We used one-hot encoding for extraction of sequence-derived characteristics from the cancer type-specific and normal m^{6}A sites identified in cancer cell lines and their tumor originating healthy tissues, respectively. A deep convolutional network was then used for model development. For each cancer type-specific model, the dataset was divided into a training set, a validation set, and a testing set with an 8:1:1 ratio. As shown in Figure 2, 8 out of 12 cancer types only achieved very limited prediction performance close to a random guess of area under the receiver operating characteristic curve (AUROC) of 0.5. Although lymphoma achieved the best performance of AUROC of 0.78, the integration of genome-derived knowledges still enhances the prediction performance. In addition to one-hot encoding, we tested several other sequence-encoding approaches and consistently obtained poor performance results (Table S1). Consequently, the decision was made to rely on one-hot encoding for its ability to encompass all the sequence-derived information theoretically required for the task at hand. Taken together, the results (see Figure 2) showed that changes in m^{6}A methylation in cancer cannot be effectively distinguished at sequence level (with an average AUROC = 0.60), tested on the independent testing datasets from 12 cancer types, demonstrating that using solely sequence-based signatures fails to capture the heterogeneity and complexity of the m^{6}A transcriptomic variations across various cancers.

Cancer-induced m^{6}A alterations exhibit distinct genomic signatures and are shared across different cancer types

To search for common signatures of m^{6}A changes in cancer, we sought to train interpretable deep neural networks capable of distinguishing m^{6}A alterations between cancer and normal contexts by adding genome-derived knowledge. Given that the formation and various functions of the m^{6}A methylome are intrinsically associated with specific transcript regions or characteristics, it is reasonable to assume that leveraging this layer of information should contribute to distinguishing and, more importantly, interpreting the m^{6}A cancer epitranscriptome. Consequently, 54 additional genome-derived features were extracted and combined with the sequence information above for model development. Although genomic features only already achieved significant improvements in prediction accuracy compared with sequence-based models, the optimal performances were achieved when combining sequence inputs and genomic features tested on all 12 cancer types (Table S2), demonstrating that distinct genome differences indeed exist between cancer and normal m^{6}A epitranscriptomes. After incorporating genomic features, the proposed deep neural models improved by 8%–36% in AUROC (Figure 3A and Table S3). Specifically, the brain cancer model achieved the best improvement by 36% (sequence only model: AUROC = 0.51; integrated model: AUROC = 0.87), while the lymphoma model only improved by 8% (sequence only model: AUROC = 0.78; integrated model: AUROC = 0.86). It may be worth noting that, in previous works, sequence-based models can achieve a predictive performance of around 0.80 in AUROC for *in silico* m^{6}A identification,[10,33] indicating that m^{6}A and non-m^{6}A sites can be effectively classified at the sequence level. However, our results revealed that, given the precondition that both positive and negative samples are experimentally validated m^{6}A sites, the dynamic changes of m^{6}A

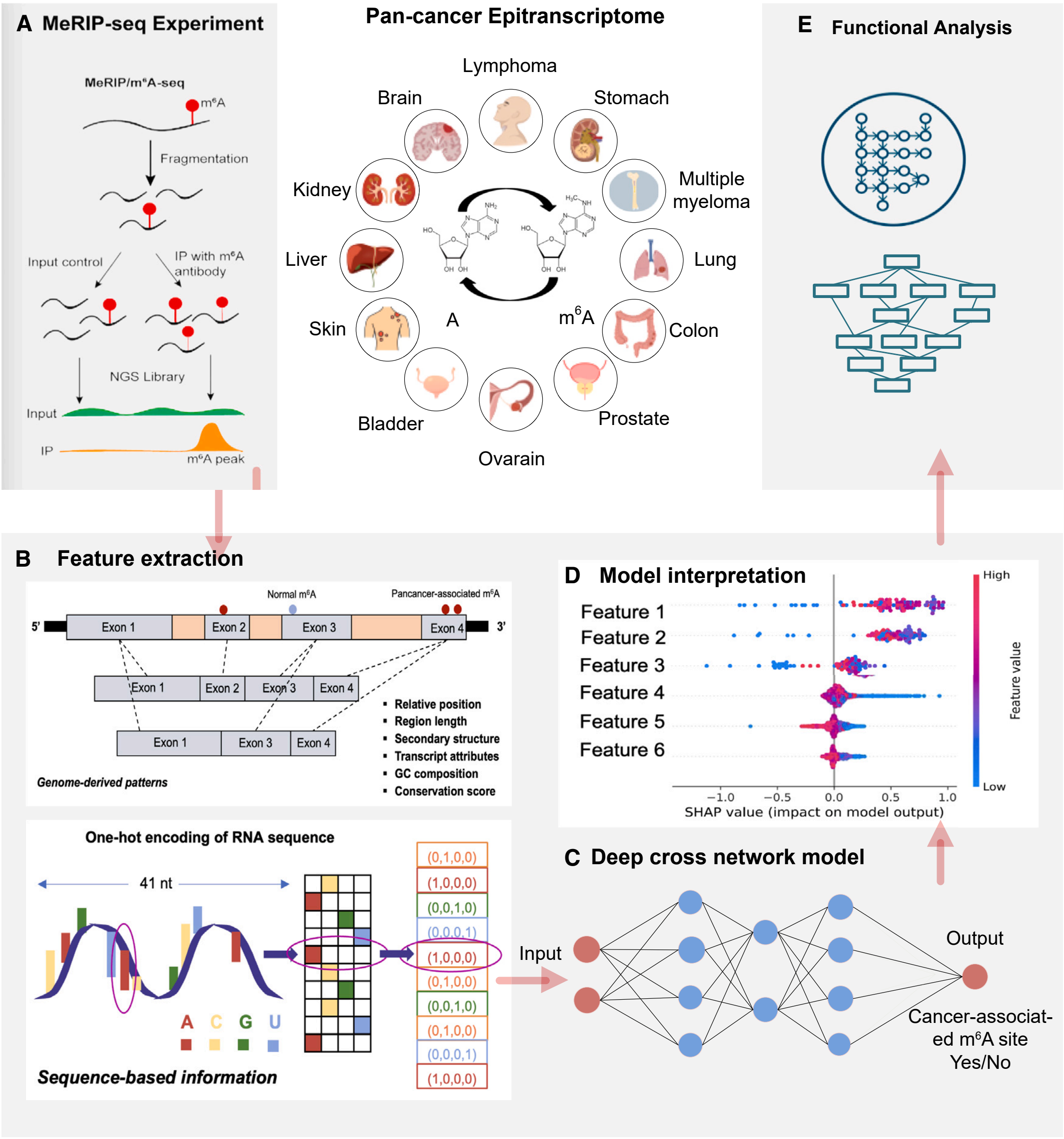

Figure 1. Overall design of the study centered on pan-cancer epitranscriptomes

(A) The epitranscriptome datasets corresponding to 12 cancer types and their respective normal control samples were obtained from public databases. The m^{6}A epitranscriptomes under cancer and normal contexts were extracted by a standard MeRIP-seq data analysis pipeline. (B and C) The sequence-based and genome-derived features were extracted for in-depth analysis by deep learning approaches for predicting the cancer-associated m^{6}A methylation. (D) The Shapley additive explanations (SHAP) were applied for model interpretation to gain insights from the trained deep learning models by identifying the genomic landmarks that significantly contribute to the prediction of cancer-associated m^{6}A sites across multiple cancer types. (E) Gene ontology enrichment and pathway analysis were conducted to understand the common functional impacts of cancer-associated epitranscriptome disturbance.

methylation in cancers primarily rely on genome-derived features for effective classification.

Of interest here is exploring whether shared patterns of m^{6}A alternations exist across different cancer types. To this end, we conducted cancer type cross-testing on 12 cancer type-specific models. Specifically, a model trained on one cancer type was independently tested on the other cancer-specific datasets. Intriguingly, we observed that several categories of cancer type-specific models exhibited a certain degree of clustering (Figure 3B), and sometimes a model trained on an m^{6}A dataset from one individual cancer could effectively identify the m^{6}A changes in other cancer types (Figure 3C), suggesting the

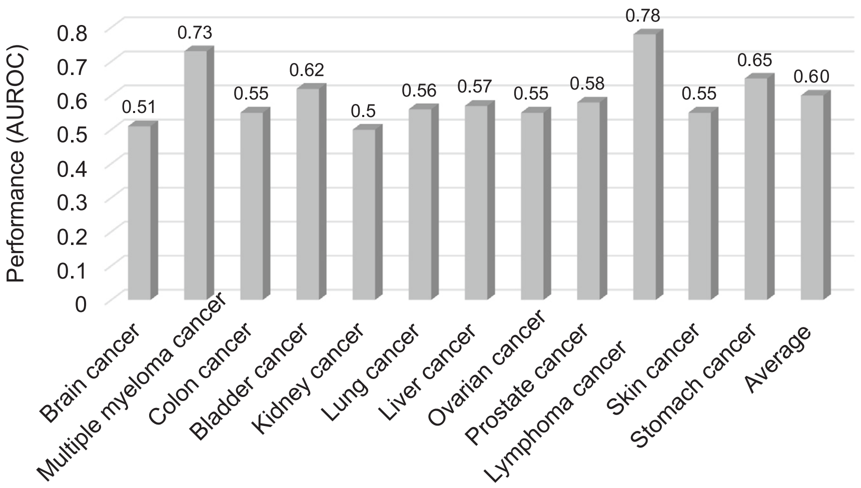

Figure 2. Performance evaluation of 12 cancer type-specific model using only sequence-based information

The AUROC was used to evaluate performance for each cancer type-specific prediction model.

existence of shared genomic signatures in m^{6}A alterations across different cancers. As shown in Figure 3B, stomach, brain, lung, prostate, colon, skin, lymphoma, and liver cancers are clustered and strongly related to one another. Taking colon cancer as an example, the model trained to identify the m^{6}A changes in colon tumors demonstrated robust performance when independently tested on six different cancer types (lung cancer: AUROC = 0.79; brain cancer: AUROC = 0.90; prostate cancer: AUROC = 0.72; liver cancer: AUROC = 0.70; skin cancer: AUROC = 0.76; stomach cancer: AUROC = 0.77). In addition, the network clustering diagrams revealed a strong connection between bladder and myeloma cancer. Meanwhile, we also found that the cancer type-specific m^{6}A patterns were indeed observed in kidney and ovarian cancer. Please refer to Table S4 for the complete results of cancer type cross-testing. Taken together, the above results indicated a certain association among the cancer type-specific models constructed based on genome-derived features, suggesting that shared genomic signatures may exist in m^{6}A alternations across various cancer types.

A generalized deep learning model for the pan-cancer analysis of m^{6}A epitranscriptomic disruptions

In the cancer type cross-testing, we observed a certain level of correlation in the genomic landscape of m^{6}A alterations across different cancer types. To better profile the shared key genomic features across different cancer types, we employed an interpretable deep learning model to capture the m^{6}A alterations among cancer contexts. To the best of our knowledge, this represents the first effort to explore the genomic characteristics of m^{6}A epitranscriptomic alterations in the pan-cancer landscape. The pan-cancer model was developed by incorporating m^{6}A sites that were simultaneously observed in most cancer conditions (but rarely in normal tissues) and those commonly present in normal tissues (but rarely in cancer conditions) (please refer to materials and methods for details). Consistent with previous cancer type-specific analyses, we found that sequence information alone could not capture the variations of m^{6}A in pan-cancer scenarios (AUROC = 0.56). We also adjusted relevant parameters, selecting different values for layers, epoch, and activation function, and tested various combinations, all resulting in AUROCs within the range of 0.50–0.55 (Table S5), further proving that sequence alone is nearly incapable of distinguishing differences. Once again, the integration of genome-derived knowledge significantly enhanced the model performance (AUROC = 0.84) (Figure S1). It may be worth noting that the incorporation of genomic features typically results in approximately a ∼10% improvement in the model performance for distinguishing between modified and non-modified residues,[34] compared with a sequence-based model. The results of our pan-cancer analyses revealed that functionally relevant m^{6}A sites may, in general, exhibit highly significant genomic characteristics (e.g., specific genomic positions) rather than variability in the RNA sequences, and our model demonstrated performance comparable with models distinguishing m^{6}A from non-m^{6}A sites. This allows us to further investigate the driving features behind the model decisions by taking advantage of advanced interpretation methods. In addition, our initial analysis with simpler models such as CNN, logistic regression, random forest, and gradient boosting showed comparable AUROC performance (Table S6). We opted for the DCN due to its ability to flexibly extract interactive features and its significantly improved computational efficiency during SHAP (Shapley additive explanations) interpretation.

Interpretation of deep learning models uncovers shared epitranscriptomic signatures in pan-cancer

Given the performance of the proposed pan-cancer model, we aimed to delve deeper into identifying the most important features behind the model decision, and thereby reveal the common signatures of m^{6}A alterations across various cancer types. Specifically, we employed SHAP to obtain the order of feature contributions, allowing us to understand how genome-derived information impacted the model's label prediction, and whether it had a positive or negative effect. We took a closer look at the top 10 most important genomic features identified in the pan-cancer model (Figure 4). The top 2 ranked features are both under the category "Relative position on the region," representing the relative position of the m^{6}A residues within the coding sequence (CDS) and 3′ untranslated region (UTR), respectively (Figure 4A). The value was calculated by dividing the distance to the 5′ end of the transcript by the length of the entire transcript, hence ranging from 0 to 1. Figure 4B shows the detailed direction (positive or negative) of feature contribution. The color bar, ranging from blue to red, signifies the feature values from low to high, while the SHAP value on the x axis indicates the positive or negative impact on the model prediction for

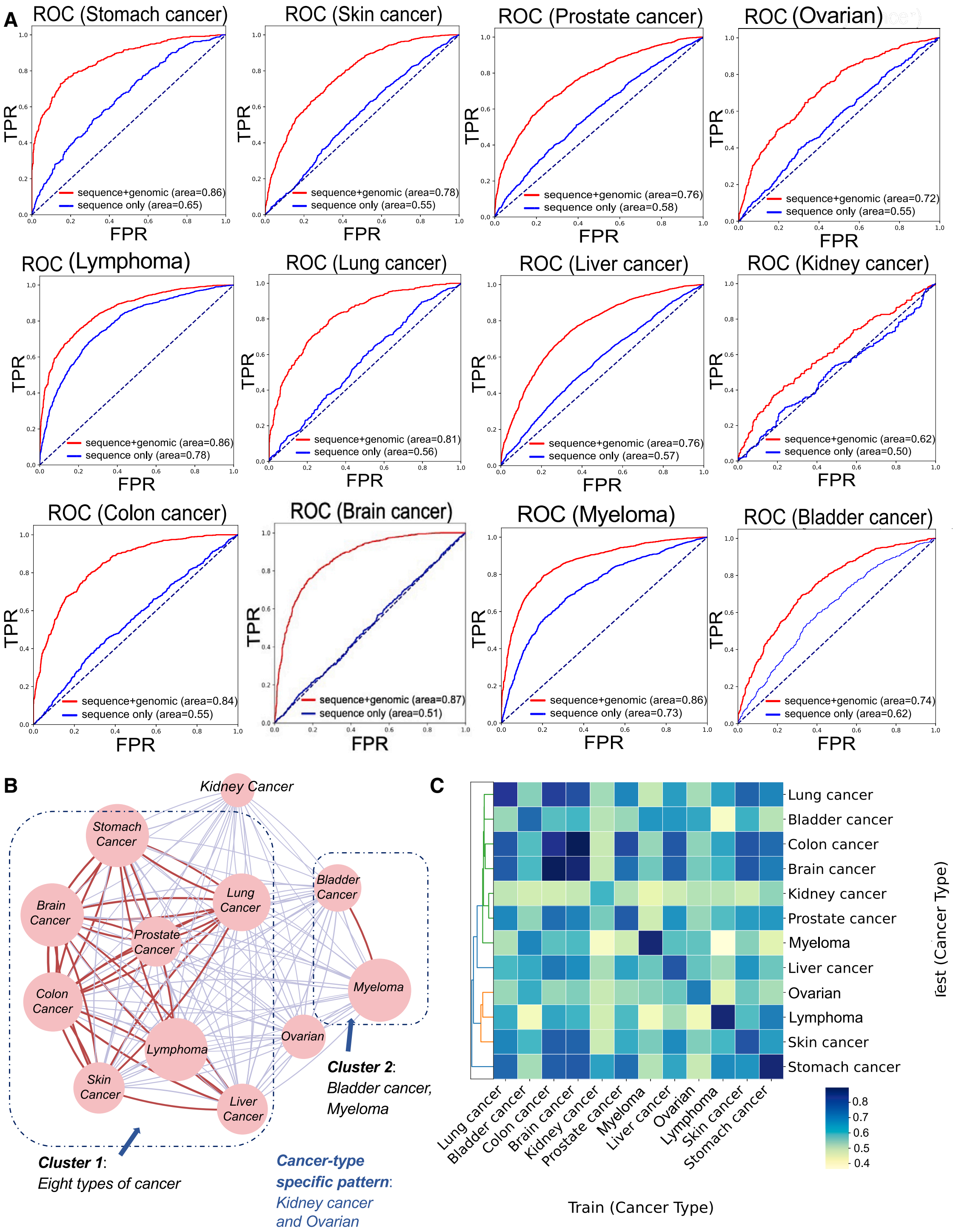

Figure 3. Performance evaluation for the integrated model and cancer type cross-testing

(A) Performance evaluation (AUROC) of sequence-based only and integrated models for 12 types of cancer-specific models. (B and C) Cancer type cross-testing was performed on 12 cancer type-specific models; a model trained on one cancer type was independently tested on the datasets of other cancer types (AUROC). The color range from gray to red in (B) represents the strength of AUROC in model testing, with gray indicating weaker performance and red indicating stronger performance. The size of the nodes indicates the degree of clustering within each cancer type. Larger nodes represent clusters with tighter interrelations among samples, reflecting a higher degree of association within the respective cancer type.

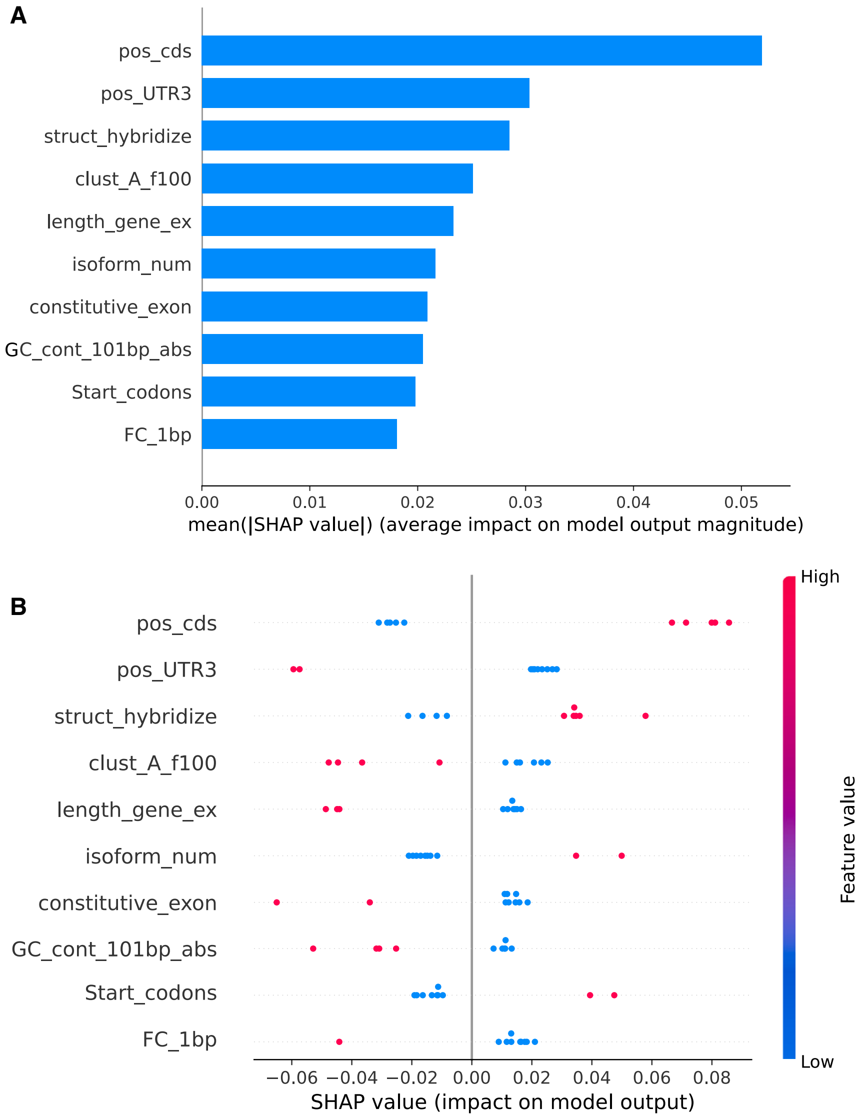

Figure 4. Model interpretation using SHAP

(A) The features are sorted by the sum of SHAP value magnitudes, the bar plot shows the absolute SHAP values of the top 10 most important genomic features for model decision. (B) The beeswarm plot shows how genome-derived information impacts the model's label prediction, whether it has a positive or negative effect, respectively (positive values indicate positive effects, and negative values indicate negative effects). The color bar, ranging from blue to red, signifies the feature values from low to high.

pan-cancer-associated m^{6}A sites. As showed in Figure 4B, within the CDS region, high values are clustered at the positive axis, whereas in the 3′ UTR, the result is precisely the opposite. The combination of these two results suggests that the pan-cancer-associated m^{6}A sites are predominantly located at the boundary between CDS and 3′ UTR, near the stop codon. These results indicated that functionally relevant m^{6}A sites closely associated with cancers are more likely to occur in critical regions of the transcript, such as the stop codon, and may function by affecting a series of biological processes (BPs), including RNA translation and splicing.

Next, we categorized the top 10 important features according to the distribution of their impact on model output, a positive impact means that the model gives greater weight to these feature values in the prediction of pan-cancer-associated m^{6}As. Three genomic features, namely "struct_hybridize," "isoform_num," and "start_codon" showed positive correlation with the model's prediction. Specifically, struct_hybridize represents whether the m^{6}A sites are overlapped with RNA hybridized regions.[35] We observed that m^{6}A sites closely associated with multiple cancers are more likely to occur in RNA hybridized regions, suggesting that dysregulated m^{6}A methylation may influence the stable formation of complementary base pairs in RNA molecules.[36] For instance, mRNA codons may mispair with tRNA anticodons through RNA hybridization, leading to the incorporation of incorrect amino acids during protein synthesis. Previous studies have indicated that abnormal splicing of mRNA isoforms may be closely associated with diseases and cancer initialization.[37] Our pan-cancer model indicated that m^{6}A sites associated with cancers tend to be located in transcripts with greater isoform diversity, suggesting that m^{6}A modification may induce the generation of diverse isoforms through aberrant splicing, and these isoforms could potentially play distinct roles in the development and progression of various cancer stages. In addition, we observed that the region containing the start codon also contributes significantly and positively to the model's prediction of pan-cancer-associated m^{6}A sites. Considering previous findings, such as the regulatory role of oncogenic circRNA with an m^{6}A-modified start codon in the mechanism of oncogene activation in cancer,[38] it would be intriguing to further explore the molecular mechanism of 3′ UTR m^{6}As in cancers.

We next sought to interpret the pan-cancer neural model by identifying features that, when the feature value increases, tend to decrease the likelihood of a positive prediction by the model. Specifically, we observed that pan-cancer-associated m^{6}A sites were generally not enriched in constitutive exons but were enriched in alternative exons (feature: constitutive_exon). This observation aligns with the interpretation of feature "isoform_num" mentioned before. It is well known that, in cancer cells, splicing mechanisms may undergo

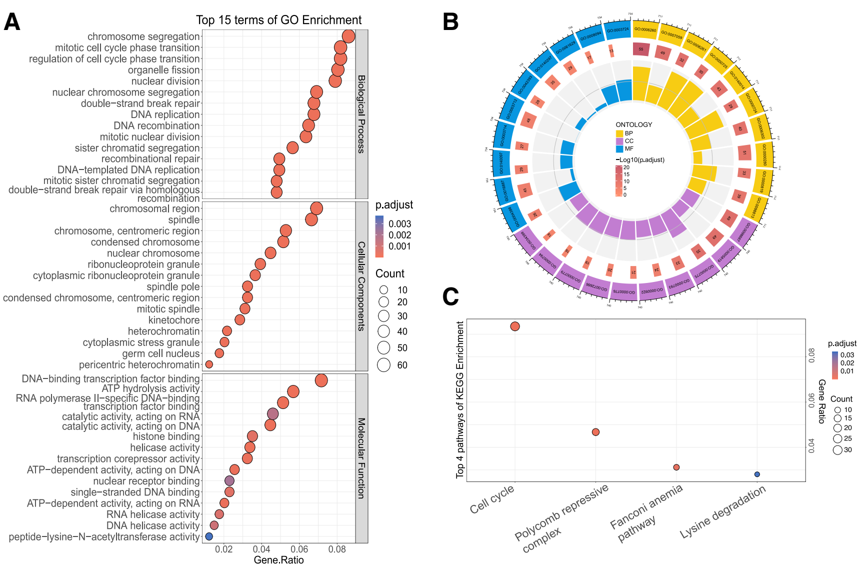

Figure 5. Functional characterization of the most pan-cancer-associated m^{6}A methylome

(A and B) The top 15 results of gene ontology (GO) enrichment analysis including biological process (BP), cellular component (CC), and molecular function (MF), respectively. (C) Kyoto Encyclopedia of Genes and Genomes (KEGG) analysis shows the enriched pathways for genes that host pan-cancer-associated m6A sites.

abnormalities, leading to the variations in alternative exons and isoform diversity. Taken together, these findings suggested that m^{6}A methylation may influence the RNA splicing mechanism, particularly the selective splicing of alternative exons (m^{6}A modifications may occur in regions closely associated with splicing events), leading to the generation of mRNA isoforms in cancer cells that may exhibit abnormal protein structures and functions, thereby promoting cancer development.

Functional characterization of m^{6}A methylome involved in the pan-cancer landscape

To gain further insights into the potential mechanisms of m^{6}A methylation in cancer regulation, especially across various cancer types, we examined the putative functional relevance of genes hosting pan-cancer-associated m^{6}A sites using gene ontology (GO) enrichment and Kyoto Encyclopedia of Genes and Genomes analysis. Figures 5A and 5B show the top 15 results of GO enrichment analysis in BP, cellular component (CC), and molecular function (MF), respectively. Specifically, we found that the pan-cancer-associated m^{6}A-hosting genes were mainly enriched on BPs related to cell cycle (chromosome segregation, $p = 8.24E-19$; nuclear chromosome segregation, $p = 7.43E-16$; DNA replication, $p = 9.67E-19$; mitotic nuclear division, $p = 4.66E-16$). These results were consistent with previous findings indicating that m^{6}A modification is closely associated with cell-cycle progression in multiple cancers.[39–41] The enriched CCs were also correlated with the mitotic cell cycle, which can be indicated by terms such as chromosomal region ($p = 9.33E-15$), spindle ($p = 2.67E-12$), ribonucleoprotein granule ($p = 4.90E-07$). Besides, many of the enriched MFs have also been confirmed to be related to m^{6}A in recent studies, including gene expression regulation[42] (DNA-binding transcription factor binding, $p = 2.37E-11$; transcription corepressor activity, $p = 7.19E-07$), transcription[43] (helicase activity, $p = 2.29E-09$; RNA helicase activity, $p = 9.70E-06$; DNA helicase activity, $p = 1.69E-05$). Meanwhile, genes hosting pan-cancer-associated m^{6}A sites were indeed significantly enriched in pathways relating to cancer development (Figure 5C), including the cell division in cancer progression[44,45] (cell cycle, $p = 8.09E-14$), regulation of gene expression, especially in cell differentiation and embryonic development (polycomb repressive complex, $p = 4.0142E-07$), DNA damage response and repair[46,47] (Fanconi anemia pathway, $p = 2.565E-05$), and tumor cell metabolism[48] (lysine degradation, $p = 5.08E-4$). Please refer to Tables S7 and S8 for the complete results.

DISCUSSION

m^{6}A RNA methylation functions as a core regulator in cellular signaling pathways, playing a pivotal role in many essential BPs, such as RNA stability, splicing, and translation. As a dynamic regulatory mechanism, dysregulated m^{6}A modification is widely involved in tumor processes from proliferation to metastasis.[4,49] Acknowledging the significant role of genomic characteristics to the development of DNA methylation-based cancer biomarkers, we performed a pan-cancer analysis, for the first time, to systematically explore the shared genomic patterns of alterations in m^{6}A methylation across 12 distinct cancer types. This may contribute to generating novel insights relevant to cancer therapy, especially in comprehending regulatory mechanisms through the epitranscriptome layer.

Leveraging an extensive collection of m^{6}A profiling datasets derived from cancer cell lines and their corresponding tumor-originating normal tissues, we trained 12 distinct cancer type-specific deep cross network models. These models successfully distinguish between specific pairs of normal and cancer m^{6}A contexts using genome-based features. However, it is important to acknowledge the proposed model's limitation in confidently classifying non-specific methylation sites (m^{6}A sites without significant differences between cancerous and normal conditions). This limitation is due to the fact that our model was trained using strongly cancer-associated and normal m^{6}A residues.

Interestingly, cross-cancer type testing indicated the existence of shared genomic patterns across various cancers at epitranscriptome layer, and finally a pan-cancer model was developed to identify the shared patterns that could not be observed in a single cancer type. Compared with cancer type-specific models, the pan-cancer model

(AUROC = 0.86) achieved varying improvements in prediction performance across different cancer types (Figure 3A). It notably improved predictions for kidney cancer (AUROC = 0.62) and ovarian cancer (AUROC = 0.72), while showing similar performance for brain cancer (AUROC = 0.87) and stomach cancer (AUROC = 0.86). These variations may be attributed to shared genomic patterns observed across different cancer types (Figures 3B and 3C), with these common genomic features likely driving the predictions. Consequently, our study aims to interpret the pan-cancer model to identify shared and significant epitranscriptomic signatures across cancers.

The interpretation of deep cross network models has enabled us to better comprehend the impact of genome-derived features on model's decision. We found that the pan-cancer-associated m^{6}A sites were generally more enriched in the intersecting region of the CDS and 3′ UTR, specifically near the stop codon, compared with m^{6}A sites found in normal tumor-originating tissues. Considering that important cellular processes, such as transcriptional silencing, are often regulated by the methylation of a small segment within a single region of a functional element, it becomes evident how crucial these specific modifications can be.[50,51] Thus, exploring shared patterns and precisely identifying the location of changes in the m^{6}A epitranscriptome should contribute to understanding RNA methylation-based biomarkers, particularly across various cancer types. Besides, through our analysis of the driving features, we observed that struct_hybridize ranked the top 3 most important feature with high positive attribution values in identifying pan-cancer-associated m^{6}A sites. This finding suggested that pan-cancer-associated m^{6}A sites are likely to occur within RNA hybridized regions. Changes in m^{6}A regulation on these regions may be involved in the pairing of mRNA codons with tRNA anticodons through RNA hybridization,[36] a process particularly crucial for subsequent protein synthesis. In addition, studies have demonstrated that tumor cells typically exhibit aberrant splicing of mRNA isoforms, impacting key BPs such as cell-cycle regulation, apoptosis, invasion, and metastasis.[52] These aberrant splicing events contribute to the initiation and progression of cancer. Our analysis highlights a close association between pan-cancer-associated m^{6}A sites and alternative exons. Meanwhile, pan-cancer-associated m^{6}A-enriched transcripts tend to exhibit more isoform diversity. These results highlighted the association between aberrant m^{6}A methylation patterns in alternative splicing and cancer pathogenesis. For example, certain mRNA isoforms generated through m^{6}A-regulated alternative splicing may be specific to particular cancer stages.

In addition, the distribution plots suggested that cancer type-specific m^{6}A sites were generally enriched in the front region of the CDS in 8 out of 12 cancer types, particularly in kidney, ovarian, stomach, and bladder cancers (Figure S2). Furthermore, we also observed an enrichment change in the 5′ UTR for cancer type-specific m^{6}A sites in five cancer types: bladder, brain, liver, lung, and colon cancers. Interestingly, this trend was reversed in lymphoma, while the remaining six cancer types showed no significant differences. Besides the cancer type-specific dataset, we then performed an enrichment change analysis of four types of m^{6}A modification sites. We first examined pan-cancer-associated and normal tissue-associated m^{6}A sites, followed by non-specific methylation sites (m^{6}A sites without significant differences between cancerous and normal conditions) and all m^{6}A sites (Figure S3). Generally, we observed that pan-cancer-associated and normal tissue-associated m^{6}A sites were significantly enriched in the end CDS and 3′ UTRs compared with the patterns for non-specific and all m^{6}A sites. Upon closer inspection, pan-cancer-associated methylation sites generated a slightly higher peak than that of normal tissue-associated m^{6}A methylation sites. In the front and middle CDS regions, pan-cancer-associated methylation sites were more enriched closer to the middle CDS region, while normal tissue-associated m^{6}A sites showed a peak in the front CDS region. In addition, the overall patterns of non-specific m^{6}A sites and all m^{6}A sites exhibited very similar trends.

In summary, gaining a deeper understanding of the shared genomic signatures formed by diverse cancer types through m^{6}A alterations is essential for unraveling the molecular mechanisms that drive cancer progression. We hope that our findings, after further experimental validation, may contribute new insights into m^{6}A-regulated mechanisms in cancer, potentially aiding in the development of diagnostic procedures and targeted therapeutic strategies. These may include but are not limited to potential biomarkers based on m^{6}A alterations occurring in specific transcript regions, RNA-targeted therapies related to certain m^{6}A clusters, and the use of m^{6}A alterations as prognostic markers that are specific to certain stages of cancer progression.

MATERIALS AND METHODS

Data collection and processing

To try to uncover the shared patterns of m^{6}A alternations across cancers, we collected a large array of m^{6}A epitranscriptome profiles from various resources. Specifically, the experimentally validated base-resolution m^{6}A sites were extracted from the m^{6}A-Atlas v.2.0 database.[53] We filtered the original collection to retain only m^{6}A sites identified in at least 2 independent studies, resulting in 134,038 high-confidence m^{6}A sites identified at base-resolution level. Next, the dynamic m^{6}A alternations in cancers were obtained from 167 m6A-seq datasets. We first extracted m6A-seq datasets of normal human tissues from a comprehensive study,[54] which encompasses the most complete collection of high-quality m6A-seq datasets across human tissues in a single study. Subsequently, we identified cancer datasets by matching them with the normal tissues through an extensive literature review targeting m6A-seq samples published in highly impactful journals. In total, the context-specific m^{6}A-containing regions were extracted from 23 cancer cell lines and 12 cancer-originating normal tissues (12 cancer types in total, Table S9). The raw sequencing data of m6A-seq samples were downloaded from the Gene Expression Omnibus (GEO) repository[55] of the National Center for Biotechnology Information (NCBI)[56] and the National Genomics Data Center.[57] We employed a unified pipeline to identify the m^{6}A-enriched regions as follows. Firstly, the adaptors and reads of low quality were trimmed using Trim Galore,[58] followed by quality assessment

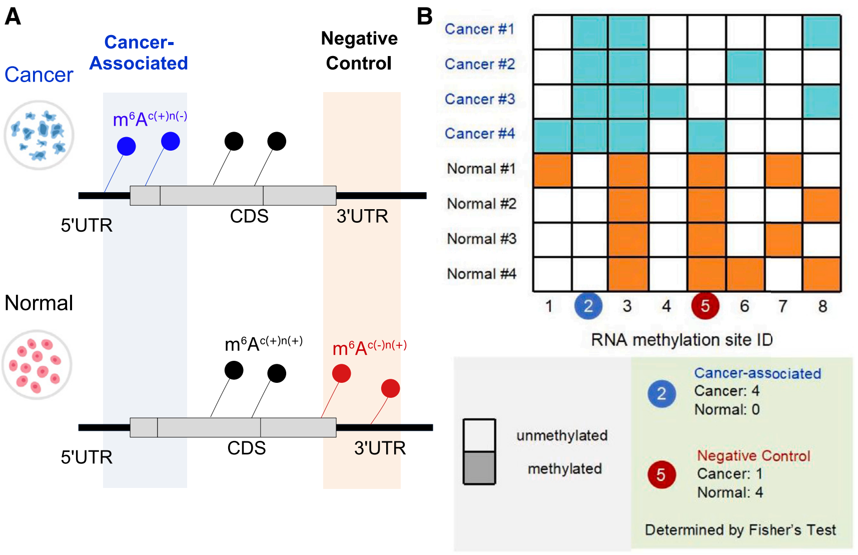

Figure 6. Selection of cancer-associated and negative control m^{6}A sites

(A) For a specific cancer type, cancer-associated m^{6}A sites are defined as the m^{6}A sites that detected in cancer but not in the corresponding normal control condition. Negative control sites were not shown under cancer conditions but appeared in the normal conditions. (B) Under a pan-cancer context, cancer-associated m^{6}A sites are those that appear more frequently under cancer conditions compared with normal control conditions, which is quantitatively determined by Fisher's exact test with a combined cutoff of $\log_2$(odds ratio) > 2 and adjusted p value < 0.05.

using FastQC. We used HISAT2[59] to align the processed reads to the human reference genome (GRCh37), followed by the peak-calling process using exomePeak2 with default setting.[60] Subsequently, all identified m^{6}A-enriched regions underwent filtration to retain peaks featuring at least one DRACH m^{6}A consensus motif. Finally, a total of 424,469 and 130,197 m^{6}A-enriched regions were obtained in cancer and normal contexts, respectively. All the processed m^{6}A epitranscriptome profiles were collected and used to train the deep neural models for subsequent analysis.

Construction of cancer-specific and pan-cancer datasets

In this study, we performed two types of analyses by developing cancer type-specific and pan-cancer models. These interpretable deep neural models were then used to explore detailed patterns and associations among different cancer types. To this end, the following selection strategies were employed.

Dataset for a single cancer type

For a specific cancer, positive data (cancer-associated m^{6}A sites) were derived by identifying m^{6}A sites that appear under cancer conditions but not in its corresponding normal tissue, and is denoted as m^{6}A$^{c(+)n(-)}$ sites. Conversely, negative data were defined by m^{6}A sites that do not exist in cancer cells but appeared in the normal tissue, and defined as m^{6}A$^{c(-)n(+)}$ sites (see Figure 6A). These methodological approaches were employed uniformly across 12 pairs of cancer type-specific datasets to ensure consistent and rigorous analysis for each individual cancer type (Table S10).

Pan-cancer datasets

Under a pan-cancer context, cancer-associated m^{6}A sites are those that appear more frequently under cancer conditions compared with normal control conditions, which was quantitatively assessed by Fisher's exact test. The p value was corrected using the Benjamini-Hochberg technique.[61] Significantly related loci were identified by filtering results with $\log_2$(odds ratio) > 2 and p < 0.05. Specifically, a total of 2,655 pan-cancer-associated and 29,812 normal m^{6}A sites were identified (Tables 11 and S12), respectively. Subsequently, the 2,655 pan-cancer-associated m^{6}A residues were selected as positive data, and 2,655 negative sited were randomly selected from 29,812 normal m^{6}A sites to keep a 1:1 positive to negative ratio.

Feature encoding approaches

One-hot encoding for sequence-based information

To obtain the sequence-derived information of targeted m^{6}A residues, the widely used one-hot encoding approach was utilized. Each nucleotide (A, C, G, U) is represented in the form of a binary vector (A [1, 0, 0, 0], C [0, 1, 0, 0], G [0, 0, 1, 0], U [0, 0, 0, 1]). Once the sequence information has been analyzed and transformed into binary vectors, the data are converted into PyTorch tensors. The transformation of the data is important to facilitate its processing through PyTorch, a widely utilized deep learning framework. To optimize the loading of data in segmented portions, DataLoader objects are instantiated for the training set, validation set, and test set. In particular, the DCN explicitly applies feature crossing at each layer, requires no manual feature engineering, and adds negligible extra complexity to the deep neural network model.[62]

Genome-derived information

Previous studies have suggested that genome-derived information such as genomic location, gene attributes, and other relevant characteristics play a dominated role in distinguishing functionally important elements from the relatively "passenger" ones.[27,63] In this study, 52 genomic (domain) features were extracted for both cancer-associated and normal m^{6}A sites. The first 13 features were constructed as dummy variables, which are binary indicators (1 or 0) indicating whether the m^{6}A sites overlap with the topological regions of the RNA transcript (e.g., 3′ UTR, 5′ UTR, CDS): only the primary (longest) transcripts for each gene were retained to eliminate any ambiguity arising from transcript isoforms. Genomic features 14–19 captured the lengths of multiple region types. Clustering information was considered in features 20–25, such as counting of adjacent

input sites and neighboring adenine. Features 26–29 included the PhastCons[64] and fitCons[65] scores for measuring evolutionary conservation of targeted m^6A sites.[64] Features 30 and 31 were considered by paying attention to the RNA secondary structures around the m^6A sites, which were predicted by the RNAfold package.[35] We next considered the attributes of the m^6A-containing genes or transcripts such as being housekeeping genes or sncRNA, as well as whether the targeted modified residues fall within the binding regions of important m^6A regulators (features 32–44). Features 45–48 represented the genomic properties (e.g., number of isoforms, GC composition) of the m^6A-containing transcripts. Finally, the relative position of targeted m^6A on multiple region types was represented by features 49–52. Please refer to Table S13 for more details about the genome-derived features considered in our model.

Model design

A DCN model specifies distinct layers and dimensions. The DCN comprises an embedding layer, a deep network module, and a cross network module. The model undergoes several training iterations using training data over a period of time. The function component of this modality comprises the embedding layer, the deep network, the crossover network, the combined output, and the activation function. The embedding layer applies a linear transformation to the input data and maps them to a higher-dimensional space defined by the embedding dimension. The deep network component consists of a series of linear layers with ReLU activation functions specifically designed to capture intricate correlations within the data. The final layer of the sequence compresses the output to a single dimension. The crossover network component processes the embedded inputs and is specifically designed to effectively represent feature interactions, with a particular focus on capturing pairwise interactions. The combined output is obtained by summing the outputs of the deep and cross networks. Finally, the activation function employs a sigmoid function to the merged output, giving a prediction within the range of [0, 1]. This prediction is utilized for the binary classification task of making predicted judgements. The architecture of the DCN facilitates the acquisition of profound feature representations and precise feature interactions, hence allowing it to proficiently execute intricate prediction tasks. The overall workflow for model design is given in Figure 7.

Embedding layer

$$x_{\text{embed}} = W_{\text{embed}} \cdot x + b_{\text{embed}} \qquad \text{(Equation 1)}$$

where x is the input, W_{embed} is the weight matrix of the embedding layer, b_{embed} is the bias term, and x_{embed} is the embedded output.

Deep layers are typically a series of transformations:

$$h_{\text{deep}} = \text{ReLU}\left(W_{\text{deep}} \cdot h_{\text{prev}} + b_{\text{deep}}\right) \qquad \text{(Equation 2)}$$

where W_{deep} and b_{deep} are the weights and biases of each layer in the deep network, and h_{prev} is the output from the previous layer (or the embedded input for the first layer).

Cross layer

$$x_{\text{cross}} = W_{\text{cross}} \cdot (x_{\text{embed}} \odot x_{\text{embed}}) + b_{\text{cross}} + x_{\text{embed}} \qquad \text{(Equation 3)}$$

where W_{cross} and b_{cross} are the weights and bias of the cross layer. The operation $\odot$ denotes element-wise multiplication.

The output, which is a vector of raw scores from the previous neural network layers, is processed using a sigmoid activation function to do binary classification:

$$y_{\text{pred}} = \sigma(\text{out}) \qquad \text{(Equation 4)}$$

where σ denotes the sigmoid function and y_{pred} is the predicted probability.

Model optimization

The training process is an essential component of developing a machine learning model, and the training process in this code may be precisely divided into the following steps. To begin, the initial step involves initializing the model, defining the loss function and optimizer, and creating instances of the DCN model using the provided hyperparameters, namely input dimension, embedding dimension, and hidden dimension. A DCN model was proposed by integrating both sequence- and genome-derived characteristics, with the following parameters: embedding dimension of 128, hidden dimension of 64, learning rate of 0.001, 10 epochs, and a batch size of 64. The loss function used is binary cross entropy loss, which is commonly employed for binary classification applications. In the optimization process of our model, the Adam optimizer was selected as the function optimizer and the learning rate for this optimizer was specifically set. The Adam optimizer is a highly efficient gradient descent algorithm that is well suited for managing extensive datasets and parameters.

The second step is the training cycle, which determines the number of training cycles required for a complete iteration over the entire training set. During each iteration of training, we reset the optimizer's gradient cache to zero. This is a common procedure to prevent the accumulation of gradients. The input data are subsequently fed into the model, which then calculates the predictions. To assess the model's performance, we computed the discrepancy between the predicted outcomes and the actual labels by utilizing a specified loss function. This step is crucial for evaluating the accuracy of the model. Following this, we applied the backpropagation algorithm to compute the gradients of the model parameters. This method is essential for understanding how to adjust the parameters to minimize the loss. Finally, we employed the optimizer's step function to update the model's weights based on these calculated gradients. This process iteratively refines the model's weights to enhance its predictive accuracy. The outcome can be computed and displayed as the mean loss across the full training dataset.

After each training cycle, we validate the model's performance using unseen data to ensure that it can generalize well. The model is

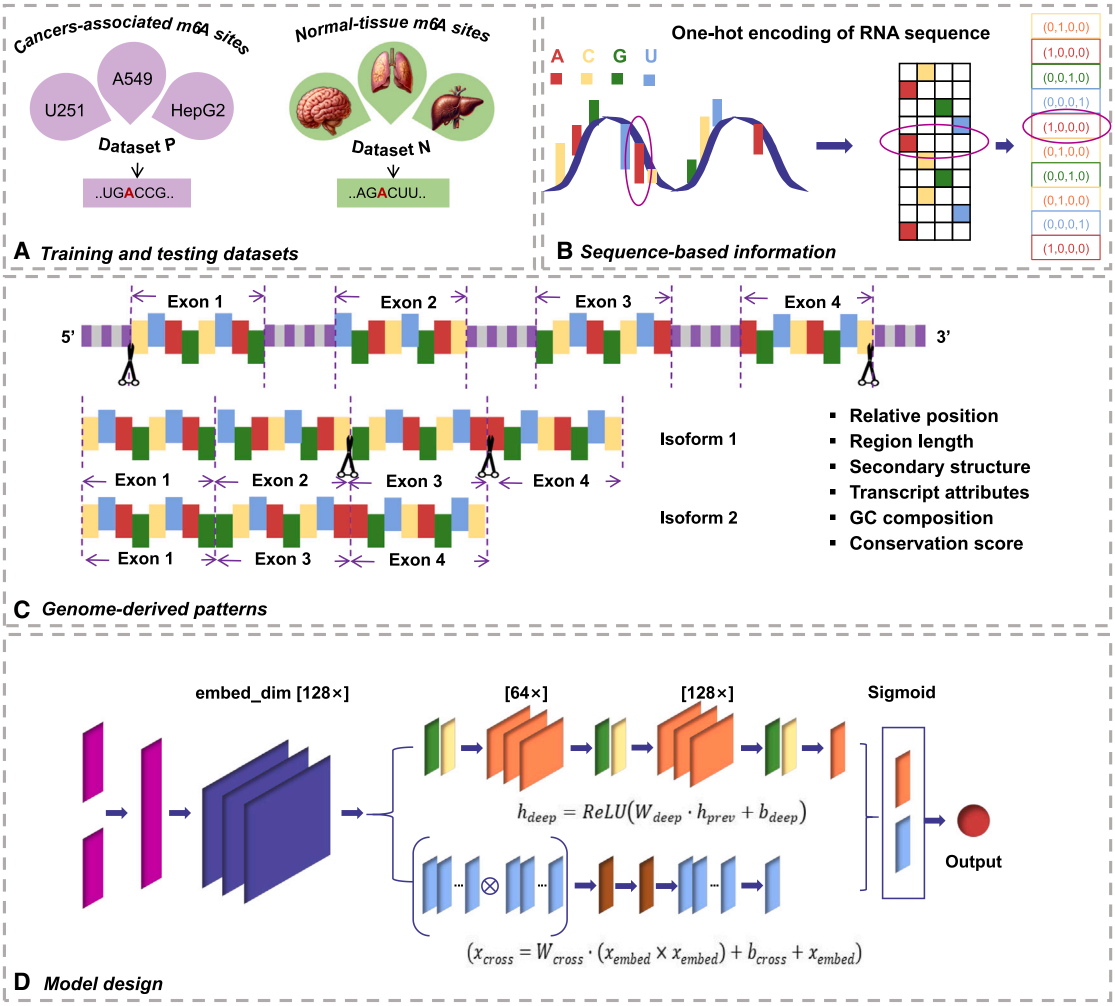

Figure 7. The overall workflow for model design

(A) Dataset preparation. (B) The 41 nt genome sequence centered on each m^6A sites was extracted, one-hot encoding strategy was used to obtain the sequence-level information. (C) Extraction of genome-derived features (Table S13). (D) A DCN model was proposed by integrating both sequence and genome-derived characteristics.

switched to evaluation mode to disable training-only functions such as dropout, ensuring a fair performance assessment. Gradient computation is also disabled to save resources, as we do not update the model during validation. We measure the model's accuracy by comparing its predictions against actual labels using a loss function, and we monitor the training and validation losses to track progress over time.

The training utilizes binary cross entropy loss for binary classification tasks, which is defined as follows:

$$\text{BCELoss}(y, \hat{y}) = -[y \log(\hat{y}) + (1 - y)\log(1 - \hat{y})] \quad \text{(Equation 5)}$$

where y is the true label (0 or 1) and $\hat{y}$ is the probability (between 0 and 1) predicted by the model.

Gradient descent is an optimization approach employed to minimize the loss function. The model parameters are updated using a specific equation:

$$\theta = \theta - \eta \nabla_\theta J(\theta) \quad \text{(Equation 6)}$$

in this equation, θ is the model parameters, η is the learning rate, $\nabla_\theta J(\theta)$ represents the loss function with respect to the parameter θ.

The Adam optimizer updates model weights through a process involving several steps and parameters:

$$\theta_{t+1} = \theta_t - \eta \frac{\widehat{m_t}}{\sqrt{\widehat{v_t}} + \epsilon} \quad \text{(Equation 7)}$$

in which values $\widehat{m_t}$ and $\widehat{v_t}$ represent the estimations of the first- and second-order moments of the gradient, which correspond to the momentum and scale terms, respectively. The small constant ϵ is introduced to ensure numerical stability, and η represents the learning rate.

In the training of neural networks, the gradient is calculated via backpropagation, encapsulated succinctly by the chain rule of calculus. The binary cross entropy loss function serves as a metric for evaluating the discrepancies in binary classification.[66]

Evaluation metrics

In the PyTorch framework, we activated the model's evaluation mode to assess its performance. This step is particularly crucial for specific layers such as dropout and batch normalization, as their behavior differs between training and evaluation phases. Activating the evaluation mode ensures that these layers function correctly for performance assessment. In addition, this mode prevents the computation of gradients, thus reducing memory usage and accelerating the evaluation process, as adjustments to model parameters are not required during this phase. In our study, key metrics were used to evaluate the binary classification performance of the DCN model. These include the true positive rate (TPR), false positive rate (FPR), and the AUROC.

The TPR, sometimes referred to as sensitivity or recall, is formally defined as:

$$\mathrm{TPR} = \frac{\mathrm{TP}}{\mathrm{TP+FN}} \quad \text{(Equation 8)}$$

TP represents the count of true classes, which refers to the number of positive cases that the model correctly predicts. FN, on the other hand, represents the count of false negative classes, which refers to the number of positive instances that the model mistakenly predicts.

The FPR quantifies the ratio of negative instances that are erroneously classified as positive by the model. It is formally expressed as:

$$\mathrm{FPR} = \frac{\mathrm{FP}}{\mathrm{FP+TN}} \quad \text{(Equation 9)}$$

TN is the number of true negative classes and FP is the number of false positive classes (the number of negative cases that the model mistakenly predicted) (the number of negative instances correctly predicted by the model).

ROC curves are graphed in a two-dimensional space, with the x axis representing the FPR and the y axis representing the TPR. The AUROC value, ranging from 0 to 1, quantifies the model's overall classification performance, with higher values indicating greater accuracy. An AUROC value of 0.5 suggests random guessing, while 1.0 indicates perfect classification. The DCN model exemplifies a systematic method for addressing binary classification tasks, especially beneficial in situations involving sequence data and necessitating comprehension of feature interactions. The implementation demonstrates efficient data preparation, model building, training methodologies, and evaluation approaches, resulting in a comprehensive evaluation of the model's prediction skills.[67]

Model interpretation

To identify the key features driving the classification of pan-cancer-associated and normal m^6A methylomes, we employed SHAP for interpretation of a deep learning model.[68] Specifically, the SHAP method "The DeepExplainer" is a specialized tool in the SHAP library that is specifically developed for interpreting deep learning models. This explainer employs the notion of SHAP values, which are based on game theory, to elucidate the results of machine learning models.[69] Within the realm of deep learning, where models are frequently intricate and comprise numerous layers, the DeepExplainer offers a means to comprehend the impact of each input characteristic on the model's predictions. SHAP values, including those computed by DeepExplainer, are derived from the principle of Shapley values in cooperative game theory. The formula for determining the Shapley value of a feature, denoted as i, in a model with N features, is as follows:

$$\phi_i(v) = \sum_{S \subseteq N \setminus \{i\}} \frac{|S|!(|N| - |S| - 1)!}{|N|!}(v(S \cup \{i\}) - v(S)) \quad \text{(Equation 10)}$$

where $\phi_i(v)$ is the Shapley value for feature i, N is the total number of features, S is a subset of features excluding feature i, $|S|$ is the number of features in subset S, $v(S)$ is the prediction (or value) of the model with the input features in set S, $v(S \cup \{i\})$ is the prediction of the model with the input features in set S plus feature i.

When interpreting the variables mentioned above, the Shapley value $\phi_i(v)$ for a certain feature i represents the average impact of feature i on the difference in model predictions, considering both the presence and absence of that feature. The summation is computed over all potential subsets of features denoted as S that do not include the specific feature i. The factor $|S|!(|N| - |S| - 1)!/|N|!$ is the weight assigned to the number of permutations of features, where feature i can be included in the model after the features in set S. The term $v(S \cup \{i\}) - v(S)$ represents the marginal contribution of feature i when added to the set S.

Computing this formula directly in deep learning models poses a challenge due to the large number of features and the intricate nature of the model. DeepExplainer uses sampling and perturbation techniques to estimate the computation of these values, hence simplifying the model's behavior in relation to important inputs. Although the computation of Shapley values in deep models is simply an estimation, the fundamental premise remains intact: assessing the influence of each feature on the model's output. Specifically, the contribution of the feature was showed by SHAP bar plot using the average absolute SHAP value, indicating how greater the impact of the feature on the

model. Besides the bar plot, the SHAP bee swarm plot shows the distribution of the feature's effect on the model prediction, with red color representing high values. Positive SHAP values signify features that positively influence the model's prediction, while negative values suggest features that hurt the forecast.[69]

DATA AND CODE AVAILABILITY

The raw data used in this study are already publicly available in the NCBI GEO database with the detailed description (accession number) listed in Table S8. Raw data: http://www.ncbi.nlm.nih.gov. The processed data used to train and independently test the proposed deep cross model is collected in Tables S10 and S11. The complete code for data selection and model development can be found in the supplemental information.

ACKNOWLEDGMENTS

Natural Science Foundation of Jiangsu Province (grant no. BK20240723), Scientific Research Foundation for Distinguished Professor at Nanjing University of Chinese Medicine (grant no. 013038030001), National Natural Science Foundation of China (grant no. 31671373), XJTLU Key Program Special Fund (KSF-E-51 and KSF-P-02). This work is supported by the Supercomputing Platform of Xi'an Jiaotong-Liverpool University. Funding for open access charge: Natural Science Foundation of Jiangsu Province (BK20240723).

AUTHOR CONTRIBUTIONS

B.S. and J.M. defined the research's analytical approach and strategized the program. R.X. was responsible for data analysis, model establishment, and bioinformatics analysis. X.Y. conducted GO analysis. R.X. drafted the figures and manuscript. K.C., J.M., and Z.W. supervised and assisted in the analysis. B.S., J.M., D.J.R., J.S., and N.B. contributed to the manuscript revision. All authors read and approved the final version of the manuscript.

DECLARATION OF INTERESTS

The authors declare no competing interests.

SUPPLEMENTAL INFORMATION

Supplemental information can be found online at https://doi.org/10.1016/j.omtn.2024.102376.

REFERENCES

1. Rhodes, D.R., and Chinnaiyan, A.M. (2005). Integrative analysis of the cancer transcriptome. Nat. Genet. *37*, S31–S37.
2. Yi, E., Chamorro González, R., Henssen, A.G., and Verhaak, R.G.W. (2022). Extrachromosomal DNA amplifications in cancer. Nat. Rev. Genet. *23*, 760–771.
3. Song, B., Wang, X., Liang, Z., Ma, J., Huang, D., Wang, Y., de Magalhães, J.P., Rigden, D.J., Meng, J., Liu, G., et al. (2022). RMDisease V2.0: an updated database of genetic variants that affect RNA modifications with disease and trait implication. Nucleic Acids Res. *51*, D1388–D1396.
4. Han, M., Sun, H., Zhou, Q., Liu, J., Hu, J., Yuan, W., and Sun, Z. (2023). Effects of RNA methylation on Tumor angiogenesis and cancer progression. Mol. Cancer *22*, 198.
5. Yang, B., Wang, J.Q., Tan, Y., Yuan, R., Chen, Z.S., and Zou, C. (2021). RNA methylation and cancer treatment. Pharmacol. Res. *174*, 105937.
6. Song, B., Tang, Y., Chen, K., Wei, Z., Rong, R., Lu, Z., Su, J., de Magalhães, J.P., Rigden, D.J., and Meng, J. (2020). m7GHub: deciphering the location, regulation and pathogenesis of internal mRNA N7-methylguanosine (m7G) sites in human. Bioinformatics *36*, 3528–3536.
7. Wang, X., Zhang, Y., Chen, K., Liang, Z., Ma, J., Xia, R., de Magalhães, J.P., Rigden, D.J., Meng, J., and Song, B. (2024). m7GHub V2.0: an updated database for decoding the N7-methylguanosine (m7G) epitranscriptome. Nucleic Acids Res. *52*, D203–D212.
8. Sun, T., Wu, R., and Ming, L. (2019). The role of m6A RNA methylation in cancer. Biomed. Pharmacother. *112*, 108613.
9. An, Y., and Duan, H. (2022). The role of m6A RNA methylation in cancer metabolism. Mol. Cancer *21*, 14.
10. Zhou, Y., Zeng, P., Li, Y.H., Zhang, Z., and Cui, Q. (2016). SRAMP: prediction of mammalian N6-methyladenosine (m6A) sites based on sequence-derived features. Nucleic Acids Res. *44*, e91.
11. Zhang, C., Zhi, W.I., Lu, H., Samanta, D., Chen, I., Gabrielson, E., and Semenza, G.L. (2016). Hypoxia-inducible factors regulate pluripotency factor expression by ZNF217- and ALKBH5-mediated modulation of RNA methylation in breast cancer cells. Oncotarget *7*, 64527–64542.
12. Zhang, Q., and Xu, K. (2023). The role of regulators of RNA m^6A methylation in lung cancer. Genes Dis. *10*, 495–504.
13. Ni, Z., Sun, P., Zheng, J., Wu, M., Yang, C., Cheng, M., Yin, M., Cui, C., Wang, G., Yuan, L., et al. (2022). JNK Signaling Promotes Bladder Cancer Immune Escape by Regulating METTL3-Mediated m6A Modification of PD-L1 mRNA. Cancer Res. *82*, 1789–1802.
14. Zhang, L., Li, Y., Zhou, L., Zhou, H., Ye, L., Ou, T., Hong, H., Zheng, S., Zhou, Z., Wu, K., et al. (2023). The m6A Reader YTHDF2 Promotes Bladder Cancer Progression by Suppressing RIG-I-Mediated Immune Response. Cancer Res. *83*, 1834–1850.
15. Xu, Q., Ren, N., Ren, L., Yang, Y., Pan, J., and Shang, H. (2024). RNA m6A methylation regulators in liver cancer. Cancer Cell Int. *24*, 1.
16. Wang, J., Yu, H., Dong, W., Zhang, C., Hu, M., Ma, W., Jiang, X., Li, H., Yang, P., and Xiang, D. (2023). N6-Methyladenosine-Mediated Up-Regulation of FZD10 Regulates Liver Cancer Stem Cells' Properties and Lenvatinib Resistance Through WNT/β-Catenin and Hippo Signaling Pathways. Gastroenterology *164*, 990–1005.
17. Geffen, Y., Anand, S., Akiyama, Y., Yaron, T.M., Song, Y., Johnson, J.L., Govindan, A., Babur, Ö., Li, Y., Huntsman, E., et al. (2023). Pan-cancer analysis of post-translational modifications reveals shared patterns of protein regulation. Cell *186*, 3945–3967.e26.
18. Jha, A., Quesnel-Vallières, M., Wang, D., Thomas-Tikhonenko, A., Lynch, K.W., and Barash, Y. (2022). Identifying common transcriptome signatures of cancer by interpreting deep learning models. Genome Biol. *23*, 117.
19. Ghoshdastider, U., and Sendoel, A. (2023). Exploring the pan-cancer landscape of posttranscriptional regulation. Cell Rep. *42*, 113172.
20. Wei, C., Wang, B., Peng, D., Zhang, X., Li, Z., Luo, L., He, Y., Liang, H., Du, X., Li, S., et al. (2022). Pan-Cancer Analysis Shows That ALKBH5 Is a Potential Prognostic and Immunotherapeutic Biomarker for Multiple Cancer Types Including Gliomas. Front. Immunol. *13*, 849592.
21. Wei, W., Liu, C., Wang, C., Wang, M., Jiang, W., Zhou, Y., and Zhang, S. (2022). Comprehensive pan-cancer analysis of N7-methylguanosine regulators: Expression features and potential implications in prognosis and immunotherapy. Front. Genet. *13*, 1016797.
22. Shen, S., Zhang, R., Jiang, Y., Li, Y., Lin, L., Liu, Z., Zhao, Y., Shen, H., Hu, Z., Wei, Y., and Chen, F. (2021). Comprehensive analyses of m6A regulators and interactive coding and non-coding RNAs across 32 cancer types. Mol. Cancer *20*, 67.
23. Lin, Y., Li, J., Liang, S., Chen, Y., Li, Y., Cun, Y., Tian, L., Zhou, Y., Chen, Y., Chu, J., et al. (2023). Pan-cancer Analysis Reveals m6A Variation and Cell-specific Regulatory Network in Different Cancer Types. Dev. Reprod. Biol. *22*, qzae052.
24. Kuhn, A.B., Feis, D.L., Schilbach, L., Kracht, L., Hess, M.E., Mauer, J., Bruning, J.C., and Tittgemeyer, M. (2016). FTO gene variant modulates the neural correlates of visual food perception. Neuroimage *128*, 21–31.
25. Li, Y., Liu, Y., Zhang, Y., Wu, Y., Xing, Z., Wang, J., and Fan, G.H. (2023). Discovery of a First-in-Class CD38 Inhibitor for the Treatment of Mitochondrial Myopathy. J. Med. Chem. *66*, 12762–12775.
26. Chen, S., Zhang, L., Lu, L., Meng, J., and Liu, H. (2021). FBCwPlaid: A Functional Biclustering Analysis of Epi-transcriptome Profiling Data via a Weighted Plaid Model. IEEE ACM Trans. Comput. Biol. Bioinf *19*, 1640–1650.
27. Koch, A., Joosten, S.C., Feng, Z., de Ruijter, T.C., Draht, M.X., Melotte, V., Smits, K.M., Veeck, J., Herman, J.G., Van Neste, L., et al. (2018). Analysis of DNA methylation in cancer: location revisited. Nat. Rev. Clin. Oncol. *15*, 459–466.
28. Montavon, G., Samek, W., and Müller, K.-R. (2018). Methods for interpreting and understanding deep neural networks. Digit. Signal Process. *73*, 1–15.

29. Hanczar, B., Zehraoui, F., Issa, T., and Arles, M. (2020). Biological interpretation of deep neural network for phenotype prediction based on gene expression. BMC Bioinf. *21*, 501.

30. Wang, C., He, Z., Jia, R., Pan, S., Coin, L.J., Song, J., and Li, F. (2024). PLANNER: a multi-scale deep language model for the origins of replication site prediction. IEEE J Biomed Health Inform *PP*.

31. Song, Z., Huang, D., Song, B., Chen, K., Song, Y., Liu, G., Su, J., Magalhães, J.P.d., Rigden, D.J., and Meng, J. (2021). Attention-based multi-label neural networks for integrated prediction and interpretation of twelve widely occurring RNA modifications. Nat. Commun. *12*, 4011.

32. Zhang, Y., Wang, Z., Zhang, Y., Li, S., Guo, Y., Song, J., and Yu, D.J. (2023). Interpretable prediction models for widespread m6A RNA modification across cell lines and tissues. Bioinformatics *39*, btad709.

33. Chen, W., Ding, H., Zhou, X., Lin, H., and Chou, K.C. (2018). iRNA(m6A)-PseDNC: Identifying N^6-methyladenosine sites using pseudo dinucleotide composition. Anal. Biochem. *561–562*, 59–65.

34. Liu, L., Song, B., Chen, K., Zhang, Y., de Magalhães, J.P., Rigden, D.J., Lei, X., and Wei, Z. (2022). WHISTLE server: A high-accuracy genomic coordinate-based machine learning platform for RNA modification prediction. Methods *203*, 378–382.

35. Lorenz, R., Bernhart, S.H., Höner Zu Siederdissen, C., Tafer, H., Flamm, C., Stadler, P.F., and Hofacker, I.L. (2011). ViennaRNA Package 2.0. Algorithm Mol. Biol. *6*, 26.

36. Abakir, A., Giles, T.C., Cristini, A., Foster, J.M., Dai, N., Starczak, M., Rubio-Roldan, A., Li, M., Eleftheriou, M., Crutchley, J., et al. (2020). N^6-methyladenosine regulates the stability of RNA:DNA hybrids in human cells. Nat. Genet. *52*, 48–55.

37. Nikom, D., and Zheng, S. (2023). Alternative splicing in neurodegenerative disease and the promise of RNA therapies. Nat. Rev. Neurosci. *24*, 457–473.

38. Li, Y., Chen, B., Zhao, J., Li, Q., Chen, S., Guo, T., Li, Y., Lai, H., Chen, Z., Meng, Z., et al. (2021). HNRNPL Circularizes ARHGAP35 to Produce an Oncogenic Protein. Adv. Sci. *8*, 2001701.

39. Xu, W., Lai, Y., Pan, Y., Tan, M., Ma, Y., Sheng, H., and Wang, J. (2022). m6A RNA methylation-mediated NDUFA4 promotes cell proliferation and metabolism in gastric cancer. Cell Death Dis. *13*, 715.

40. Yang, Z., Wang, T., Wu, D., Min, Z., Tan, J., and Yu, B. (2020). RNA N6-methyladenosine reader IGF2BP3 regulates cell cycle and angiogenesis in colon cancer. J. Exp. Clin. Cancer Res. *39*, 203.

41. Luo, H., Liu, W., Zhang, Y., Yang, Y., Jiang, X., Wu, S., and Shao, L. (2021). METTL3-mediated m^6A modification regulates cell cycle progression of dental pulp stem cells. Stem Cell Res. Ther. *12*, 159.

42. Sendinc, E., and Shi, Y. (2023). RNA m6A methylation across the transcriptome. Mol. Cell *83*, 428–441.

43. Liu, J., Dou, X., Chen, C., Chen, C., Liu, C., Xu, M.M., Zhao, S., Shen, B., Gao, Y., Han, D., and He, C. (2020). N^6-methyladenosine of chromosome-associated regulatory RNA regulates chromatin state and transcription. Science *367*, 580–586.

44. Icard, P., Fournel, L., Wu, Z., Alifano, M., and Lincet, H. (2019). Interconnection between Metabolism and Cell Cycle in Cancer. Trends Biochem. Sci. *44*, 490–501.

45. Jamasbi, E., Hamelian, M., Hossain, M.A., and Varmira, K. (2022). The cell cycle, cancer development and therapy. Mol. Biol. Rep. *49*, 10875–10883.

46. Jackson, S.P., and Bartek, J. (2009). The DNA-damage response in human biology and disease. Nature *461*, 1071–1078.

47. Srinivas, U.S., Tan, B.W.Q., Vellayappan, B.A., and Jeyasekharan, A.D. (2019). ROS and the DNA damage response in cancer. Redox Biol. *25*, 101084.

48. Yuan, H., Wu, X., Wu, Q., Chatoff, A., Megill, E., Gao, J., Huang, T., Duan, T., Yang, K., Jin, C., et al. (2023). Lysine catabolism reprograms tumour immunity through histone crotonylation. Nature *617*, 818–826.

49. Jiang, X., Liu, B., Nie, Z., Duan, L., Xiong, Q., Jin, Z., Yang, C., and Chen, Y. (2021). The role of m6A modification in the biological functions and diseases. Signal Transduct. Targeted Ther. *6*, 74.

50. Gonzalgo, M.L., Hayashida, T., Bender, C.M., Pao, M.M., Tsai, Y.C., Gonzales, F.A., Nguyen, H.D., Nguyen, T.T., and Jones, P.A. (1998). The role of DNA methylation in expression of the p19/p16 locus in human bladder cancer cell lines. Cancer Res. *58*, 1245–1252.

51. Yoshikawa, H., Matsubara, K., Qian, G.S., Jackson, P., Groopman, J.D., Manning, J.E., Harris, C.C., and Herman, J.G. (2001). SOCS-1, a negative regulator of the JAK/STAT pathway, is silenced by methylation in human hepatocellular carcinoma and shows growth-suppression activity. Nat. Genet. *28*, 29–35.

52. Sciarrillo, R., Wojtuszkiewicz, A., Assaraf, Y.G., Jansen, G., Kaspers, G.J.L., Giovannetti, E., and Cloos, J. (2020). The role of alternative splicing in cancer: From oncogenesis to drug resistance. Drug Resist. Updates *53*, 100728.

53. Liang, Z., Ye, H., Ma, J., Wei, Z., Wang, Y., Zhang, Y., Huang, D., Song, B., Meng, J., Rigden, D.J., and Chen, K. (2023). m6A-Atlas v2.0: updated resources for unraveling the N6-methyladenosine (m6A) epitranscriptome among multiple species. Nucleic Acids Res. *52*, D194–D202.

54. Liu, J., Li, K., Cai, J., Zhang, M., Zhang, X., Xiong, X., Meng, H., Xu, X., Huang, Z., Peng, J., et al. (2020). Landscape and Regulation of m^6A and m^6Am Methylome across Human and Mouse Tissues. Mol. Cell *77*, 426–440.e6.

55. Barrett, T., Wilhite, S.E., Ledoux, P., Evangelista, C., Kim, I.F., Tomashevsky, M., Marshall, K.A., Phillippy, K.H., Sherman, P.M., Holko, M., et al. (2013). NCBI GEO: archive for functional genomics data sets–update. Nucleic Acids Res. *41*, D991–D995.

56. Wheeler, D.L., Church, D.M., Edgar, R., Federhen, S., Helmberg, W., Madden, T.L., Pontius, J., Schuler, G.D., Schriml, L.M., and Sequeira, E. (2004). Database resources of the National Center for Biotechnology Information. Nucleic Acids Res. *32*, 13–16.

57. CNCB-NGDC Members and Partners (2021). Database Resources of the National Genomics Data Center, China National Center for Bioinformation in 2021. Nucleic Acids Res. *49*, D18–D28.

58. Martin, M. (2011). Cutadapt removes adapter sequences from high-throughput sequencing reads. EMBnet. j. *17*, 10–12.

59. Kim, D., Paggi, J.M., Park, C., Bennett, C., and Salzberg, S.L. (2019). Graph-based genome alignment and genotyping with HISAT2 and HISAT-genotype. Nat. Biotechnol. *37*, 907–915.

60. Meng, J., Lu, Z., Liu, H., Zhang, L., Zhang, S., Chen, Y., Rao, M.K., and Huang, Y. (2014). A protocol for RNA methylation differential analysis with MeRIP-Seq data and exomePeak R/Bioconductor package. Methods *69*, 274–281.

61. Statology (2023). A Guide to the Benjamini-Hochberg Procedure. https://www.statology.org/tutorials/.

62. Wang, R., Fu, B., Fu, G., and Wang, M. (2017). Deep & Cross Network for Ad Click Predictions. Proceedings of the ADKDD *17*, 1–7.

63. Song, B., Chen, K., Tang, Y., Wei, Z., Su, J., de Magalhães, J.P., Rigden, D.J., and Meng, J. (2021). ConsRM: Collection and large-scale prediction of the evolutionarily conserved RNA methylation sites, with implications for the functional epitranscriptome. Briefings Bioinf. *22*, bbab088.

64. Siepel, A., Bejerano, G., Pedersen, J.S., Hinrichs, A.S., Hou, M., Rosenbloom, K., Clawson, H., Spieth, J., Hillier, L.W., Richards, S., et al. (2005). Evolutionarily conserved elements in vertebrate, insect, worm, and yeast genomes. Genome Res. *15*, 1034–1050.

65. Gulko, B., Hubisz, M.J., Gronau, I., and Siepel, A. (2015). A method for calculating probabilities of fitness consequences for point mutations across the human genome. Nat. Genet. *47*, 276–283.

66. Goodfellow, I., Bengio, Y., and Courville, A. (2016). Deep Learning (MIT press).

67. Shan, Y., Hoens, T.R., Jiao, J., Wang, H., Yu, D., and Mao, J.C. (2016). Deep Crossing. Proceedings of the 22nd ACM SIGKDD International Conference on Knowledge Discovery and Data Mining, pp. 255–262.

68. Lundberg, S.M., Erion, G., Chen, H., DeGrave, A., Prutkin, J.M., Nair, B., Katz, R., Himmelfarb, J., Bansal, N., and Lee, S.I. (2020). From Local Explanations to Global Understanding with Explainable AI for Trees. Nat. Mach. Intell. *2*, 56–67.

69. Lundberg, S.M., and Lee, S.-I. (2017). A unified approach to interpreting model predictions. Proceedings of the 31st International Conference on Neural Information Processing Systems (Curran Associates Inc.), pp. 4768–4777.

OMTN, Volume *35*

Supplemental information

Interpretable deep cross networks unveiled common signatures of dysregulated epitranscriptomes across 12 cancer types

Rong Xia, Xiangyu Yin, Jiaming Huang, Kunqi Chen, Jiongming Ma, Zhen Wei, Jionglong Su, Neil Blake, Daniel J. Rigden, Jia Meng, and Bowen Song

Table S1. Performance evaluation using different sequence-based feature extraction approaches (AUROC)

	One-hot	K-mer counting	PSSMs
Lung cancer	0.56	0.50	0.52
Bladder cancer	0.61	0.60	0.59
Colon cancer	0.55	0.57	0.56
Brain cancer	0.52	0.50	0.52
Kidney cancer	0.51	0.50	0.52
Prostate cancer	0.59	0.58	0.50
Bone marrow cancer	0.73	0.50	0.60
Liver cancer	0.58	0.59	0.50
Ovary cancer	0.55	0.49	0.50
Lymph nodes cancer	0.78	0.64	0.59
Skin cancer	0.55	0.51	0.50
Stomach cancer	0.64	0.60	0.59

Table S2. Performance evaluation of sequence-only, genomic-only and integration models

	Sequence only model	Genomic only model	Integration model
Liver cancer	0.58	0.73	0.76
Ovary cancer	0.55	0.70	0.72
Bone marrow cancer	0.73	0.84	0.86
Bladder cancer	0.61	0.73	0.74
Colon cancer	0.55	0.83	0.84
Brain cancer	0.52	0.87	0.87
Kidney cancer	0.51	0.61	0.62
Prostate cancer	0.59	0.75	0.76
Lung cancer	0.56	0.82	0.81
Lymph nodes cancer	0.78	0.86	0.86
Skin cancer	0.55	0.78	0.78
Stomach cancer	0.64	0.83	0.81

Table S3. Performance evaluation of 12 cancer type-specific model

	Independent testing					
	Sn (%)	Sp (%)	ACC (%)	MCC	F1	AUROC
Lung cancer	0.77	0.68	0.73	0.74	0.46	0.81
Bladder cancer	0.75	0.59	0.67	0.70	0.34	0.74
Colon cancer	0.81	0.68	0.74	0.76	0.49	0.84
Brain cancer	0.80	0.75	0.78	0.78	0.55	0.87
Kidney cancer	0.51	0.61	0.56	0.54	0.12	0.62
Prostate cancer	0.62	0.68	0.65	0.64	0.29	0.76
Bone marrow cancer	0.77	0.76	0.76	0.76	0.53	0.86
Liver cancer	0.77	0.65	0.70	0.72	0.40	0.76
Ovary cancer	0.63	0.65	0.64	0.64	0.28	0.72
Lymph nodes cancer	0.75	0.77	0.76	0.76	0.53	0.86
Skin cancer	0.71	0.70	0.71	0.71	0.41	0.78
Stomach cancer	0.76	0.78	0.77	0.77	0.54	0.86

Table S4. Performance of cancer type-cross testing (AUROC)

Table S5. Performance evaluation of sequence-only input using different model settings (AUROC)

Activation Function	Two layers			Three layers			Five layers			Seven layers			10 layers		
	10 epoch	20 epoch	50 epoch	10 epoch	20 epoch	50 epoch	10 epoch	20 epoch	50 epoch	10 epoch	20 epoch	50 epoch	10 epoch	20 epoch	50 epoch
Sigmoid	0.54	0.52	0.51	0.54	0.52	0.50	0.54	0.52	0.50	0.53	0.52	0.52	0.52	0.54	0.51
ReLU	0.53	0.55	0.54	0.55	0.51	0.52	0.55	0.53	0.53	0.55	0.52	0.52	0.54	0.53	0.50
Tanh	0.54	0.52	0.52	0.54	0.50	0.51	0.52	0.52	0.50	0.54	0.52	0.50	0.54	0.50	0.52
LeakyReLU	0.50	0.51	0.53	0.53	0.50	0.52	0.54	0.52	0.52	0.52	0.51	0.52	0.55	0.52	0.53

Table S6. Performance evaluation using different algorithms

	DCN	Simple CNN	Logistic Regression	Random Forest	Gradient Boosting
Lung cancer	0.81	0.78	0.74	0.83	0.79
Bladder cancer	0.74	0.70	0.69	0.75	0.73
Colon cancer	0.84	0.78	0.76	0.82	0.79
Brain cancer	0.87	0.80	0.78	0.83	0.81
Kidney cancer	0.62	0.56	0.52	0.74	0.67
Prostate cancer	0.76	0.69	0.64	0.76	0.69
Bone marrow cancer	0.86	0.79	0.75	0.85	0.79
Liver cancer	0.76	0.70	0.63	0.83	0.68
Ovary cancer	0.72	0.67	0.61	0.74	0.69
Lymph nodes cancer	0.86	0.81	0.79	0.81	0.80
Skin cancer	0.78	0.71	0.66	0.79	0.74
Stomach cancer	0.86	0.61	0.78	0.83	0.81
Average	0.80	0.72	0.70	0.80	0.75

Table S7. Gene Ontology (GO) enrichment analysis of the most cancers-associated m6A sites involved in pan-cancer landscape

Table S8. Kyoto Encyclopedia of Genes and Genomes (KEGG) analysis of the most cancers-associated m6A sites involved in pan-cancer landscape

Table S9. m6A-MeRIP-seq samples

Table S10. Cancer type-specific dataset

Table S11. Cancers-associated m6A sites used in developing pan-cancer model

Table S12. Normal tissues m6A sites used in developing pan-cancer model

Table S13. Genomic knowledges considered in the study

ID	Name	Description	Note
1	UTR5	5' UTR	Dummy variables indicating whether the site is overlapped to the topological region on the major RNA transcript.
2	UTR3	3' UTR	
3	cds	Coding sequence	
4	Stop_codons	stop codons flanked by 100bp	
5	Start_codons	start codons flanked by 100bp	
6	TSS	downstream 100bp of TSS	
7	TSS_A	downstream 100bp of TSS on A	
8	exon_stop	exons containing stop codons	
9	alternative_exon	alternative exons	
10	constitutive_exon	constitutive exons	
11	internal_exon	Internal exons	
12	long_exon	long exons (exon length >= 400bp)	
13	last_exon	5' last_exon	
14	length_UTR3	3'UTR length	The region length in bp
15	length_UTR5	5'UTR length	
16	length_cds	coding sequence length	
17	length_tx_full	full transcript length	
18	length_gene_full	full gene length	
19	length_gene_ex	length of all exons of the gene	
20	clust_f1000	count of neighboring input site at 1001 bp	Clustering information
21	clust_f100	count of neighboring input site at 101 bp	
22	clust_A_f1000	count of neighboring A within in 2001 nt window	
23	clust_A_f100	count of neighboring A within 201 nt window	
24	dist_nearest_p2000	distance to the closest neighboring input site at 2001 bp	
25	dist_nearest_p200	distance to the closest neighboring input site at 201 bp	
26	PC_1bp	phastCons scores of the nucleotide	Scores related to evolutionary conservation
27	PC_101bp	average phastCons scores within the flanking 101 bp	
28	FC_1bp	fitCons scores of the nucleotide	
29	FC_101bp	average fitCons scores within the flanking 101 bp region	
30	struct_hybridize	predicted RNA hybridized region	RNA secondary structures
31	struct_loop	predicted RNA loop region	
32	sncRNA	sncRNA	Attributes of the genes or transcripts
33	lncRNA	lncRNA	
34	HK_genes	housekeeping genes	
35	miR_targeted_genes	miRNA targeted genes	
36	HNRNPC_eCLIP	eCLIP data of HNRNPC RNA binding sites	

37	TargetScan	predicted miRNA targeted sites by TargetScan	
38	Verified_miRtargets	miRNA targeted sites verified by experiment	
39	METTL3_TREW	overlapped with binding regions of METTL3	
40	METTL14_TREW	overlapped with binding regions of METTL14	
41	WTAP_TREW	overlapped with binding regions of WTAP	
42	METTL16_CLIP	overlapped with binding regions of METTL16	
43	ALKBH5_PARCLIP	overlapped with binding regions of ALKBH5	
44	FTO_CLIP	overlapped with binding regions of FTO	
45	isoform_num	number of isoforms	Genomic properties
46	exon_num	number of exons	
47	GC_cont_genes	GC composition of genes	
48	GC_cont_101bp_abs	GC composition of 101 bp	
49	pos_UTR5	relative position on 5'UTR	Relative position on the region
50	pos_UTR3	relative position on 3'UTR	
51	pos_cds	relative position on coding sequence	
52	pos_exons	relative position on exon	

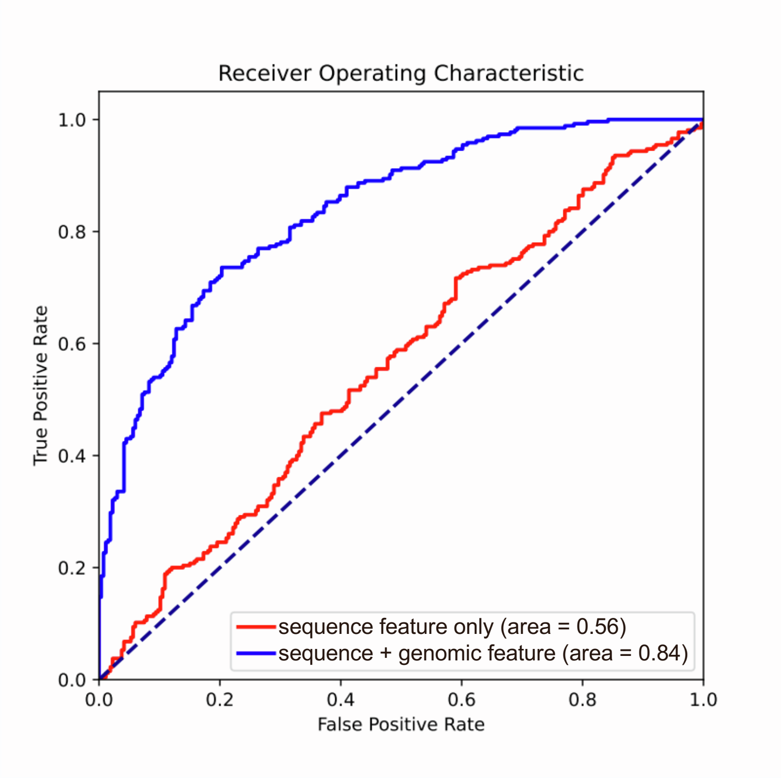

Figure S1. Performance evaluation of m^{6}A pan-cancer model (AUROC).

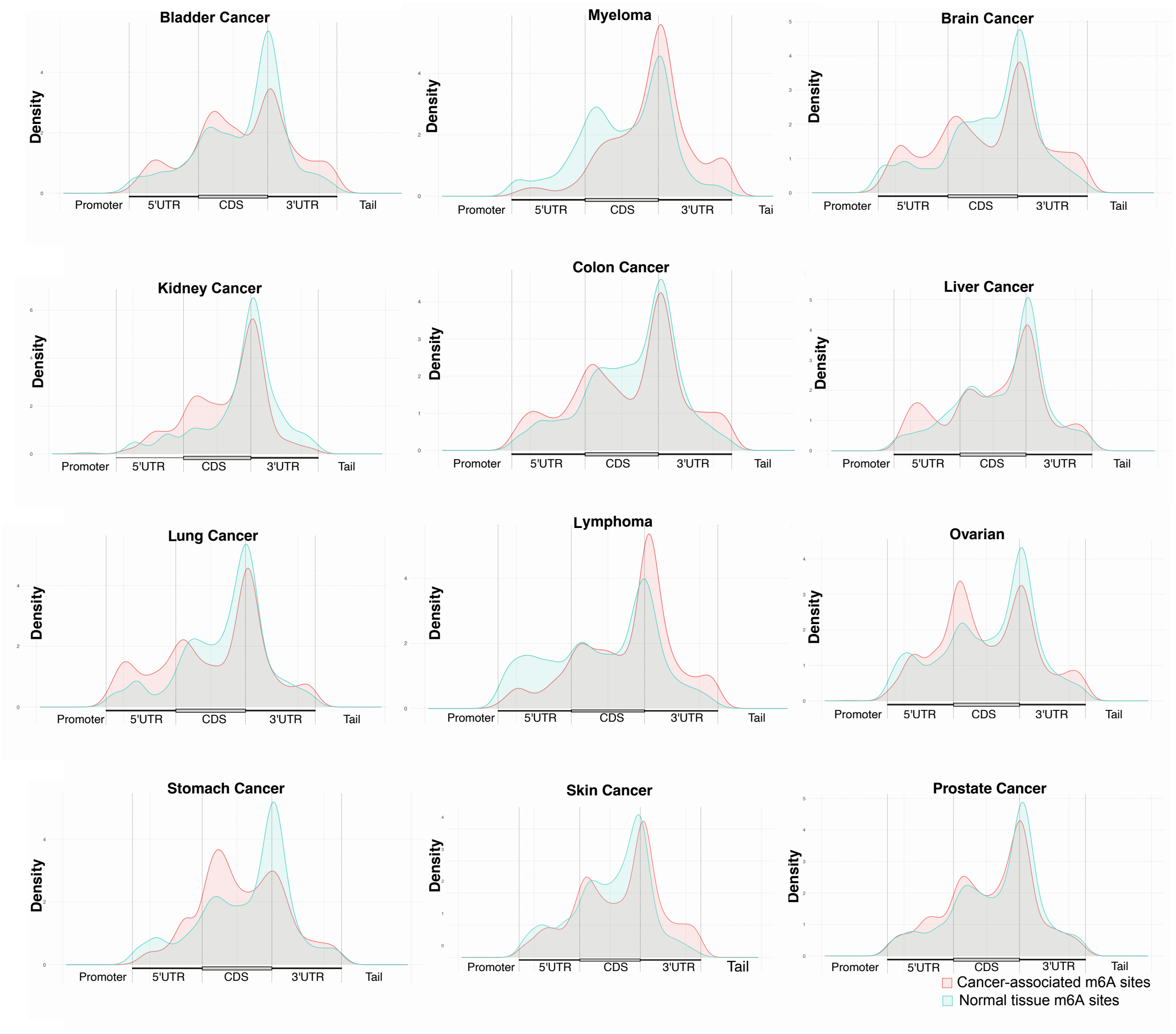

Figure S2. Distribution of 12 types of cancer type-specific m^{6}A modification sites on mRNA.

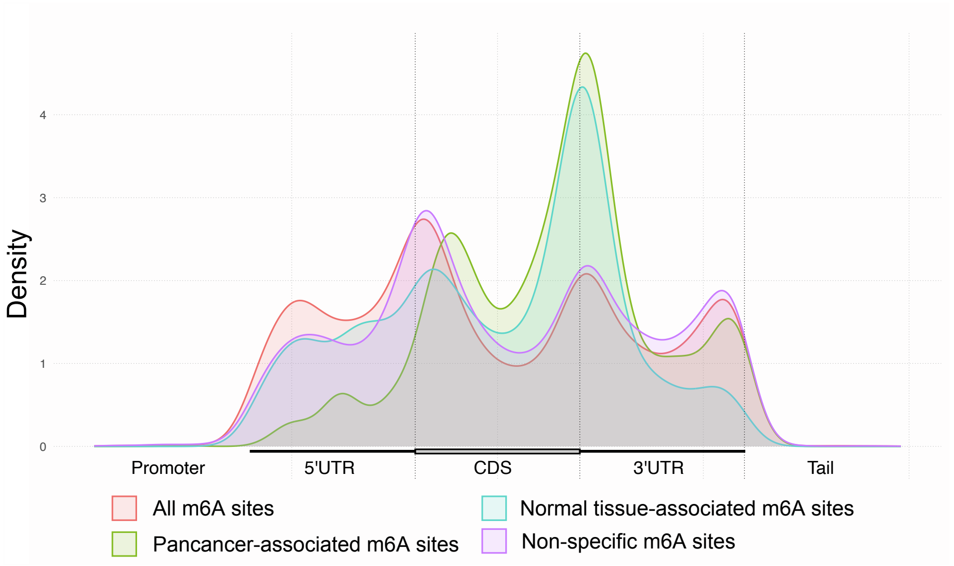

Figure S3. Distribution of pancancer-associated, normal tissue-associated, non-specific, and all m^{6}A methylation sites on mRNA.
